# A Reductive Mechanochemical Approach Enabling Direct Upcycling of Fluoride from Polytetrafluoroethylene (PTFE) into Fine Chemicals

**DOI:** 10.1021/jacs.5c14052

**Published:** 2025-10-21

**Authors:** Matthew E. Lowe, Benjamin M. Gallant, Nathan Davison, Matthew N. Hopkinson, Dominik J. Kubicki, Erli Lu, Roly J. Armstrong

**Affiliations:** † School of Natural and Environmental Sciences, 5994 Newcastle University, Newcastle Upon Tyne, NE1 7RU, U.K.; § School of Chemistry, 1724 University of Birmingham, Edgbaston, Birmingham B15 2TT, U.K.

## Abstract

Polytetrafluoroethylene (PTFE) is a highly versatile material that has found widespread application owing to its exceptionally high chemical resistance and thermal stability. However, these properties mean that PTFE disposal is an energy intensive process, producing fluorinated materials which pose serious concerns regarding toxicity and environmental persistence. Herein we report a straightforward mechanochemical approach for the reductive defluorin­ation of PTFE generating an environmentally benign mixture of elemental carbon and sodium fluoride. The process employs cheap and readily available chunks of sodium metal, proceeding rapidly at room temperature, in the absence of any organic solvent to form sodium fluoride (NaF) in 98% yield. The fluoride generated in the process can be directly upcycled into fine chemicals through in situ mechanochemical fluorin­ation reactions, delivering valuable sulfonyl fluoride and acyl fluoride products in excellent yields.

## Graphical abstract

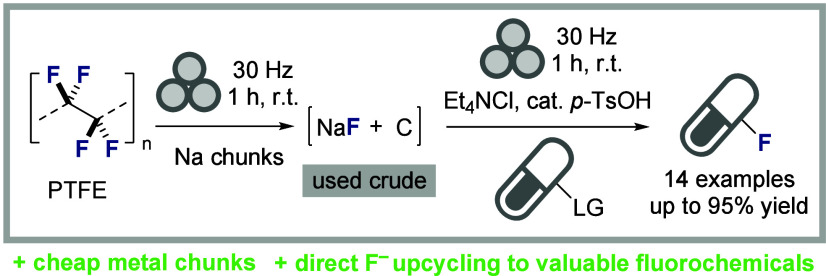

## Introduction

Polytetrafluoroethylene (PTFE), commonly known as Teflon, is a versatile fluoropolymer possessing outstanding chemical stability, exhibiting minimal reactivity with almost all chemical reagents including most mineral acids, bases and oxidizing agents (Scheme 1A).^1^ It also displays excellent thermal stability up to 260 °C, and can withstand temperatures as high as 400 °C for short durations.^2^ As a result of these remarkable properties, PTFE is used in a wide variety of applications, including household products such as nonstick coatings on cookware and membranes in textiles, as well as more specialized uses in electrical components, laboratory equipment, medical devices, lubricants, seals and gaskets. Accordingly, PTFE is produced on a very large scale (global production of 309,000 tons in 2021),^3^ but its high durability poses a major challenge at the end of its lifecycle.^4^ Disposal via landfill is problematic because PTFE does not readily biodegrade, and incineration requires very high temperatures and may release environmentally persistent perfluoroalkyl and polyfluoroalkyl substances (PFAS) which are linked to a variety of health concerns.^5^ Accordingly, there has been intense interest in approaches for chemical decomposition of PTFE, particularly methods capable of generating relatively environmentally benign inorganic fluoride salts.^6^ The majority of these defluorin­ation processes are extremely energy intensive, requiring high temperatures and forcing conditions, but elegant innovations in this area,^7,8^ including very recent work from the groups of Crimmin^9^ and Kang^10^ employing specialized magnesium reagents and electrophotocatalysis respectively, have revealed that PTFE can be defluorinated at or below room temperature. Despite these key innovations, a major limitation is that the fluorine present within PTFE is wasted. Given the intense demand for fluorinated small molecules, particularly in the pharmaceutical sector, the ideal solution would be to find a method whereby the fluorine atoms present within PTFE can be directly upcycled into valuable fluorinated fine chemicals.

**Scheme 1:**
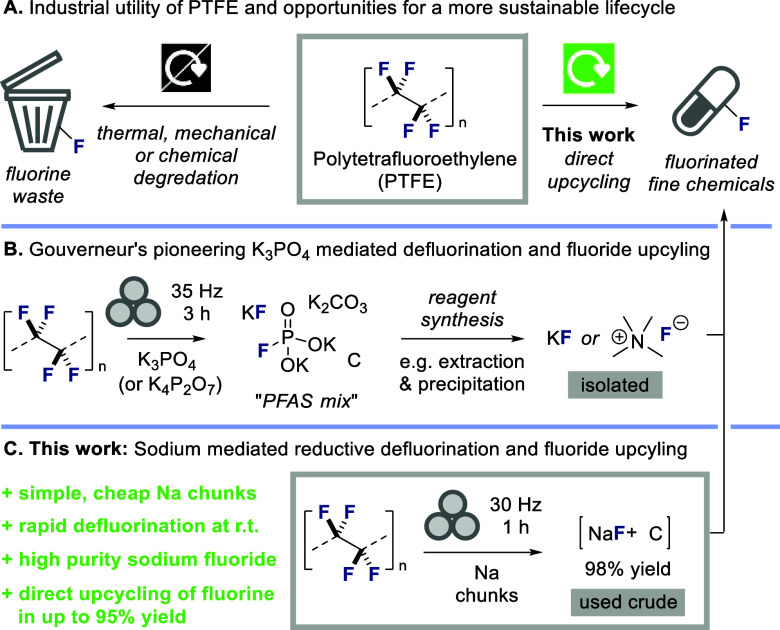
Previous Work and Strategy for the Mechanochemical Upcycling of Fluorine from PTFE into Fine Chemicals

In March 2025, Gouverneur, Paton and co-workers reported a pioneering solution to this problem, involving phosphate-mediated upcycling of PTFE (Scheme 1B).^11^ In that work, PTFE was ball milled with either K_3_PO_4_ or K_4_P_2_O_7_, leading to efficient defluorin­ation to form a so-called “PFAS mix” consisting of a mixture of fluoride, fluorophospate, phosphate, carbonate and carbon. This mixture could be transformed to fluorinating agents such as KF and tetramethylammonium fluoride which were isolated (e.g., via extraction and precipitation) and then employed in high-yielding syntheses of a variety of fluorinated organic molecules. A handful of direct fluorin­ation reactions with PFAS mix were also reported. Overall, this groundbreaking sequence of transformations enables a powerful means to capture the fluorine atoms present in PTFE and incorporate them into fluorinated fine chemicals.

Here we report a complementary solution that achieves direct upcycling of PTFE via a mechanochemical reductive defluorin­ation approach (Scheme 1C). Defluorin­ation was achieved at room temperature, by facile ball milling of PTFE with chunks of cheap, readily available sodium metal, proceeding with no organic solvent in near quantitative yield.^12^ The process delivers a simple mixture of sodium fluoride and elemental carbon, which can be directly applied (without isolation) in acid-catalyzed mechanochemical fluorin­ation reactions to obtain valuable sulfonyl fluoride and acid fluoride products in high yields. We believe that the operational simplicity of this protocol, coupled with its ability to directly deliver medicinally relevant fluorinated scaffolds, offers a practical and sustainable solution to upcycle fluorine from PTFE.

Finally, we would like to highlight several elegant, related approaches for reductive degradation of fluoropolymers, which were reported while we were finalizing this manuscript, namely: (i) solution-state reductive defluorin­ation of PTFE and other PFAS with sodium dispersion in THF reported by Shibata and co-workers;^13^ (ii) independent reports from the groups of Shibata and Ackermann involving mechanochemical degradation of fluoropolymers with KO^*t*^Bu, followed by fluoride upcycling.^14,15^ These latter studies predominantly focused upon degradation of polyvinylidene fluoride (PVDF) but were also shown to be applicable for PTFE degradation.

## Results and Discussion

We commenced our study by exploring the mechanochemical defluorin­ation of PTFE with sodium in a Retsch MM400 mixer mill employing a stainless steel milling jar with one stainless steel ball (Scheme 2A). Commercially available sodium lumps (2 equiv) were cut into approximately 5 mm pieces and were introduced into the jar, followed by addition of PTFE powder (1 equiv) and ball milling was conducted at 30 Hz for 1 h at room temperature. During milling, the external temperature of the jar was monitored using a thermocouple and was observed to rise by <2.5 °C (see Supporting Information for details). The milling jar was then opened, revealing formation of a black powder, which was analyzed via quantitative solid-state ^19^F magic angle spinning (MAS) NMR spectroscopy (Scheme 2B). We were delighted to observe near complete disappearance of the signals centered at δ_F_ = – 122.3 ppm corresponding to PTFE, along with appearance of a new signal centered at δ_F_ = – 224.2 ppm matching that of pristine NaF (see also Figure S6 and Supplementary Note 1 for a more detailed discussion). This result suggests that the reductive defluorin­ation is a remarkably clean process, with integration indicating a conversion of ≥99.9% (a critical feature for viable PTFE upcycling, given the necessity of avoiding production of smaller chain PFAS). To quantify the yield of NaF formed in this process the crude residue was agitated in water, followed by filtration to remove the black precipitate. The resulting aqueous filtrate was analyzed by quantitative solution state ^19^F NMR spectroscopy along with hexafluoroisopropanol (HFIP) as an internal standard, which indicated formation of NaF in 98% ^19^F NMR yield (Figure S7).^16^ Drying this aqueous solution under reduced pressure delivered solid NaF in 92% isolated yield. Solid-state NMR experiments support the identity and purity of this material, with ^19^F MAS NMR confirming the absence of any residual PTFE (Scheme 2B). Both this ^19^F measurement and complementary ^23^Na MAS NMR (Scheme 2C) confirm that this material is close to pure NaF, with only the presence of trace NaOH detected (likely the result of reaction between water and unreacted sodium metal).

**Scheme 2:**
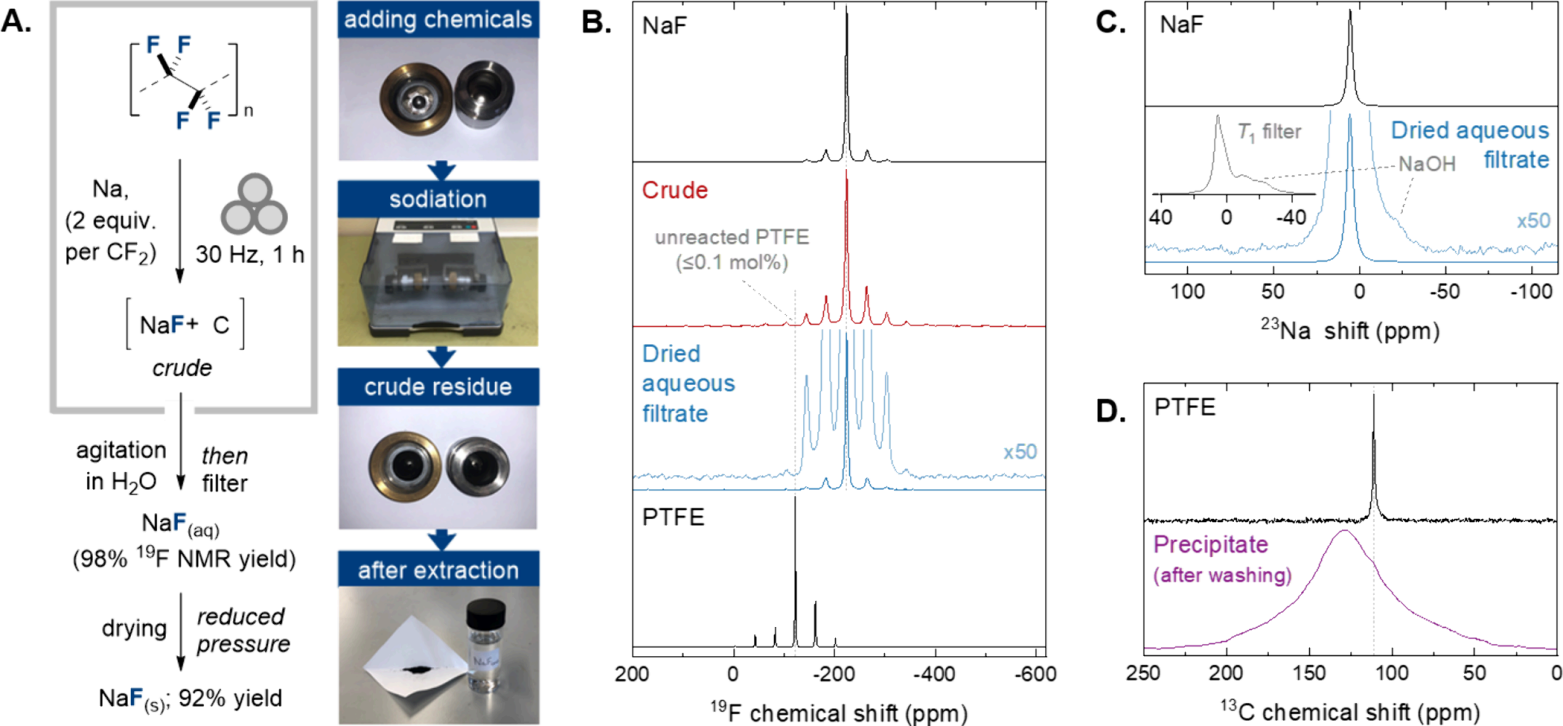
(A) Protocol for Mechanochemical Defluorination of PTFE with Sodium Metal Followed by Aqueous Extraction and Drying to Isolate NaF. (B) Quantitative Solid-State ^19^F MAS NMR Analysis (9.4 T, 15 kHz) of the Crude Residue Obtained after Mechanochemical Defluorination of PTFE with Na and the Solid Recovered by Drying of Filtrate Obtained by Washing This Residue, Alongside Reference Spectra for PTFE and NaF. (C) ^23^Na MAS NMR Analysis of the Solid Recovered by Drying of Filtrate.^a^ (D) Solid-State ^13^C MAS NMR Analysis of Pristine PTFE and Black Precipitate Recovered after Washing Crude Residue with Water

a This measurement is conducted with a single, low-tip angle pulse (0.2 μs, ∼5°) such that the spectrum is close to quantitative. Inset shows the same material recorded with a *T*_1_ filter (recycle delay, 50 ms) applied, to emphasize the faster relaxing ^23^Na nuclei in NaOH.

Detailed characterization of the black precipitate obtained after washing and filtration was also performed. ^19^F and ^23^Na MAS NMR measurements confirmed that this material contains only trace quantities of residual fluorine and sodium respectively (Figure S1–2 and S5), suggesting that the black solid is predominantly carbon-based. This hypothesis is supported by ^13^C MAS NMR, which shows a single extremely broad signal centered at δ_C_ = 132.0 ppm, suggesting a large distribution of sp^2^ carbon environments in the material, as might be expected to result after reduction of the C–F bonds of PTFE (Scheme 2D and Figure S3–4).^17^

With an efficient protocol for mechanochemical PTFE defluorin­ation established, we set out to explore direct fluoride upcycling in a subsequent mechanochemical transformation, initially focusing on the synthesis of sulfonyl fluorides, which are valuable motifs in chemical biology and covalent drug design (Table 1).^18^ Accordingly, PTFE and sodium were mechanochemically converted to a mixture of carbon and sodium fluoride under our previously optimized conditions and 4-methoxybenzenesulfonyl chloride was then introduced to the milling jar, and a further round of ball milling was performed (30 Hz, 1 h, r.t.). After this time, the milling jar was opened, and the black residue was suspended in CDCl_3_, filtered and the filtrate was analyzed by quantitative solution state ^19^F NMR analysis along with 1,1,1-trifluorotoluene (0.33 equiv) as an internal standard. Under these conditions, we were disappointed to find that the desired sulfonyl fluoride product **1a** was formed in <5% yield (entry 1). To improve the yield, inspired by recent elegant work from Kubota, Ito and co-workers on mechanochemical nucleophilic fluorin­ation of aromatic electrophiles, we explored introducing a quaternary ammonium salt (Et_4_NCl),^19^ hoping to generate a reactive tetraalkylammonium fluoride in situ, and were pleased to obtain **1a** in an improved yield of 13% (entry 2). We found that introducing a catalytic quantity (30 mol %) of a solid Brønsted acid led to a significant increase in yield, with the best result obtained with *para*-toluenesulfonic acid (*p*-TsOH), leading to formation of **1a** in 90% yield (entries 3–8). Increasing the loading of *p*-TsOH to 50 mol % delivered a further increase in yield to 96% (entry 9). We next tested various ammonium salts, all of which had a detrimental impact upon the efficiency of the fluorin­ation reaction (entries 10–14), but we were delighted to find that increasing the loading of Et_4_NCl to 2 equiv delivered the desired product **1a** in quantitative ^19^F NMR yield (entry 15). Subsequent purification by column chromatography enabled isolation of **1a** in 95% yield. A control experiment performed without Et_4_NCl afforded **1a** in a significantly reduced yield (<5%) indicating that both the ammonium salt and Brønsted acid play a key role in the fluorin­ation (entry 16).

**Table 1:**
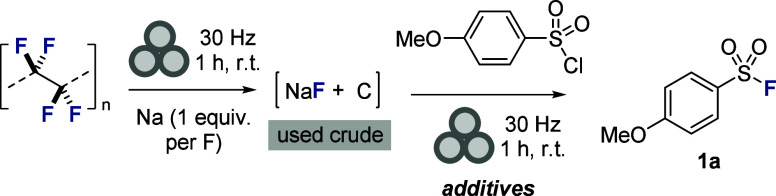
Optimization of Mechanochemical Upcycling of Fluorine from PTFE to Sulfonyl Fluorides^a^

Entry	Salt (equiv)	Acid (equiv)	Yield **1a**/%^b^
1	–	–	<5
2	Et_4_NCl (1.5)	–	13
3	Et_4_NCl (1.5)	citric acid (0.3)	67
4	Et_4_NCl (1.5)	pyridine·HCl (0.3)	34
5	Et_4_NCl (1.5)	tartaric acid (0.3)	54
6	Et_4_NCl (1.5)	PhCO_2_H (0.3)	81
7	Et_4_NCl (1.5)	CSA (0.3)	43
8	Et_4_NCl (1.5)	*p*-TsOH (0.3)	90
9	Et_4_NCl (1.5)	*p*-TsOH (0.5)	96
10	Bu_4_NCl (1.5)	*p*-TsOH (0.5)	4
11	Me_4_NCl (1.5)	*p*-TsOH (0.5)	9
12	Et_4_NBr (1.5)	*p*-TsOH (0.5)	7
13	Et_4_NI (1.5)	*p*-TsOH (0.5)	15
14	Et_4_NOTs (1.5)	*p*-TsOH (0.5)	69
15	Et_4_NCl (2.0)	*p*-TsOH (0.5)	Quant (95)
16	–	*p*-TsOH (0.5)	<5

a Reaction conditions: PTFE (2.0 equiv. based on a CF_2_ repeating unit), Na (4 equiv), ball milling at 30 Hz for 1 h at r.t. *then* 4-methoxybenzenesulfonyl chloride (1 equiv), additives (see table), ball milling at 30 Hz for 1 h at r.t.

b Yields determined by solution state ^19^F NMR analysis with 1,1,1-trifluorotoluene as an internal standard. Yields in parentheses refer to isolated material after column chromatography. CSA = (1*S*)-(+)-camphorsulfonic acid.

Having identified a highly efficient method for the mechanochemical upcycling of fluorine from PTFE into fluorochemicals, we set out to explore its generality (Scheme 3). We were pleased to find that a variety of different aromatic sulfonyl chlorides were well tolerated in this process and could be employed to access a variety of aromatic sulfonyl fluorides bearing alkoxy (**1a**), aliphatic (**1b**), aryl (**1c**), and acetamido (**1d**) groups in excellent yields (79–95%). A naphthyl analogue **1e** was isolated in 77% yield. Electron deficient sulfonyl chlorides were also evaluated, and successfully delivered the desired sulfonyl fluoride products **1f** and **1g**, albeit with somewhat diminished yields. We were also able to employ our new protocol to access aliphatic sulfonyl fluorides, with products **1h**–**j** formed in 48–54% yield. Finally, we explored expansion of the newly developed fluoride upcycling strategy to the synthesis of acyl fluorides, which are widely used intermediates in organic synthesis.^20^ Accordingly, we were delighted to find that anisoyl chloride could be transformed to anisoyl fluoride (**1k**) in 83% yield. Acyl chlorides derived from *p*-toluic acid, 4-(trifluoromethyl)­benzoic acid and *trans*-cinnamic acid delivered the corresponding acid fluorides **1l**-**n** in 73–94% yield.

**Scheme 3:**
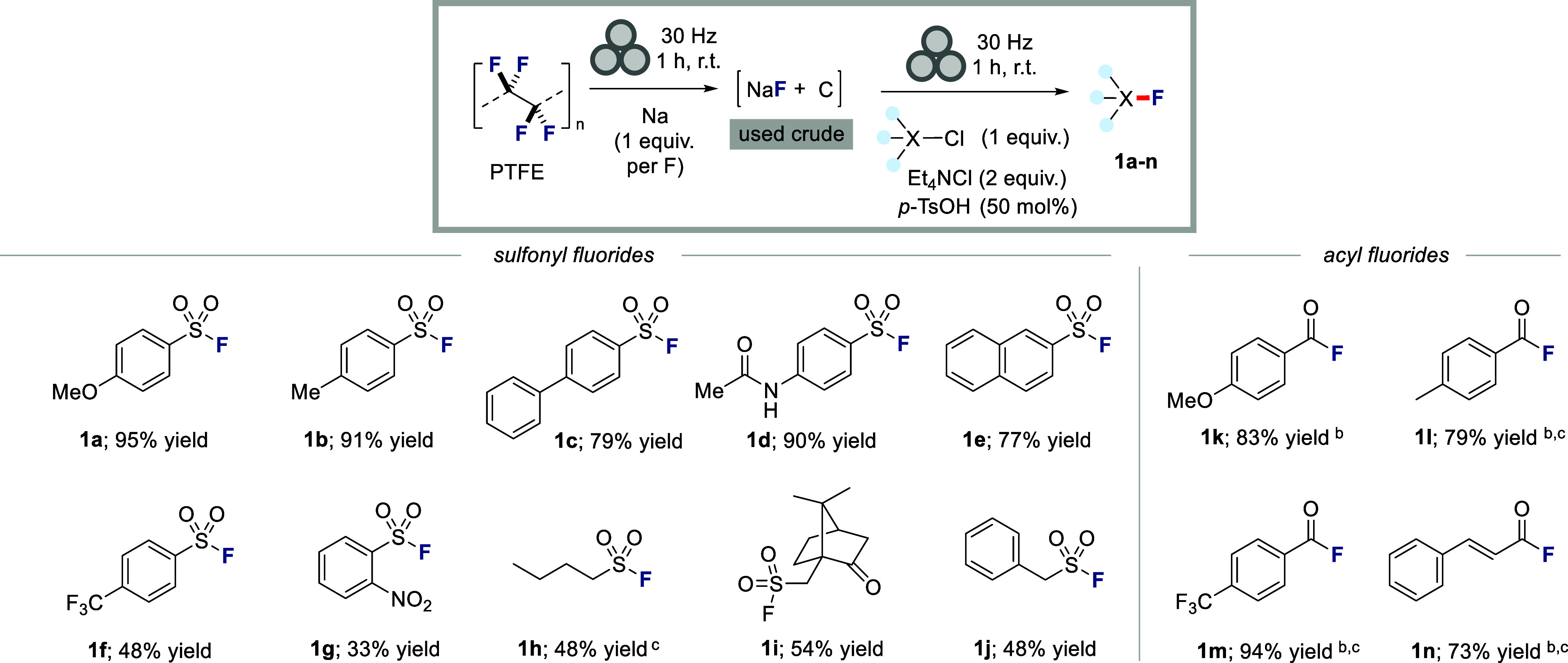
Scope of Mechanochemical Upcycling of Fluorine from PTFE to Sulfonyl Fluorides and Acid Fluorides^a^

a Reaction conditions: PTFE (2.0 equiv. based on a CF_2_ repeating unit), Na (4 equiv), ball milling at 30 Hz for 1 h at r.t. *then* sulfonyl chloride (1 equiv), Et_4_NCl (2 equiv), *p*-TsOH (0.5 equiv), ball milling at 30 Hz for 1 h at r.t. Yields refer to isolated material after column chromatography.
b Re-fluorin­ation conditions: acid chloride (1 equiv), Et_4_NCl (2 equiv), PPTS (1 equiv), ball milling at 30 Hz for 1 h at r.t.
c
^19^F NMR yields using 1,1,1-trifluorotoluene or fluorobenzene as internal standard (see Supporting Information for details).

## Conclusion

We have discovered a straightforward approach for the reductive defluorin­ation of PTFE through ball milling with commercially available, cheap lumps of sodium metal. These reactions proceed rapidly at room temperature delivering sodium fluoride in excellent efficiency (98% yield) along with an inert carbon-rich powder as the sole byproduct. The simple nature of this fluoride containing mixture enables it to be used without any purification in direct mechanochemical fluorin­ation reactions targeting valuable sulfonyl fluoride and acid fluoride products, thereby providing a direct means to upcycle fluorine from PTFE into valuable fluorinated small molecules. We believe that the operational simplicity of this protocol, combined with its ability to produce noncomplex fluoride-carbon mixtures holds substantial potential for other fluoride upcycling processes.

## Supplementary Material



## Notes

Detailed experimental procedures, characterization data for new compounds and details of computational methods (PDF)
