# Supplementary material for: A Reductive Mechanochemical Approach Enabling Direct Upcycling of Fluoride from Polytetrafluoroethylene (PTFE) into Fine Chemicals
Source: J Am Chem Soc. 2025 Oct 21;147(44):40895–9. doi: 10.1021/jacs.5c14052 (PMC12593323; doi:10.1021/jacs.5c14052)
Supplement: Supplementary file 1 [file ja5c14052_si_001.pdf]

A Reductive Mechanochemical Approach Enabling Direct Upcycling of Fluoride from Polytetrafluoroethylene (PTFE) into Fine Chemicals

Matthew E. Lowe[†], Benjamin M. Gallant[§], Nathan Davison[§], Matthew N. Hopkinson[†], Dominik J. Kubicki[§]*, Erli Lu[§]*, Roly J. Armstrong[†]*

[†] School of Natural and Environmental Sciences, Newcastle University, Newcastle Upon Tyne, NE1 7RU, UK

[§] School of Chemistry, University of Birmingham, Edgbaston, Birmingham, B15 2TT, UK

Contents

1.	General Information	S2
2.	General Procedures	S3
3.	Reductive Defluorination of PTFE	S4
3.1.	*Solid-state MAS NMR studies*	S4
3.2.	*Solution state ^{19}F NMR studies*	S15
3.3.	*Temperature studies*	S16
3.4	*Source of PTFE*	S17
4.	Upcycling of Fluoride from PTFE into Fine Chemicals	S18
4.1.	*Optimisation of reaction conditions*	S18
4.2	*Experimental procedures*	S20
4.3	*NMR spectra*	S26
5.	References	S43

1. General Information

Unless specified otherwise, all manipulations were carried out in a VigorTM glovebox equipped with a −35 ˚C freezer and a cold well, under an atmosphere of dry argon. PTFE was purchased from Thermo Fisher Scientific (catalogue number: 178930050) and dried under dynamic vacuum for several hours. Other reagents were purchased from Merck, Fluorochem or Alfa Aesar, and dried under dynamic vacuum for several hours (if solid), or dried over activated 4Å molecular sieves (if liquid), prior to use. Room temperature (r.t.) refers to 20-25 °C. Ball milling was conducted with a Retsch© MM400 mixer mill.

Flash chromatography was performed using VWR silica gel 60 (40-63 µm particle size) using head pressure by means of a nitrogen line. Sulfonyl fluoride products were purified on pre-basified silica (as noted in General Procedure A) which was prepared as follows; silica (400 g), pentane (250 mL), Et_2O (250 mL) and triethylamine (5 mL) were combined in a 1 L beaker and stirred for 15 minutes. The solvent was allowed to evaporate overnight, and the resulting silica could be stored in a sealed bottle for a few weeks.

Thin layer chromatography was performed on Merck Kieselgel 60 F_{254} 0.25 mm pre-coated aluminium plates. Product spots were visualized under UV light (λ = 254 nm) and/or by staining with potassium permanganate solution.

Solution state NMR spectroscopy was carried out using Bruker 300 MHz or 400 MHz spectrometers in the deuterated solvent stated, using the residual non-deuterated solvent signal as an internal reference. Chemical shifts are quoted in ppm with signal splitting recorded as singlet (s), doublet (d), triplet (t), quartet (q), quintet (qn), sextet (sext), septet (sept), octet (oct), nonet (non) and multiplet (m). The abbreviation br denotes broad. Coupling constants, *J*, are measured to the nearest 0.1 Hz and are presented as observed.

Solid-state ^{19}F (376.4 MHz), ^{13}C (100.6 MHz) and ^{23}Na (105.8 MHz) MAS NMR spectra were recorded on a Bruker Avance III 9.4 T spectrometer equipped with a 3.2 mm MAS probe. The powder samples were packed into 3.2 mm zirconia rotors, which were spun at a magic angle spinning rate of 15 kHz using dry nitrogen for all measurements. For ^{19}F NMR measurements a 100 kHz RF field amplitude was used and ^{19}F chemical shifts were referenced to NaF at -224.2 ppm with respect to $CFCl_3$ (0 ppm).[1] For ^{13}C NMR measurements a 71.5 kHz RF field amplitude was used and ^{13}C chemical shifts were referenced to solid adamantane at 38.48 ppm (for $-CH_2-$) with respect to tetramethylsilane at 0 ppm, in accordance with IUPAC recommendations. 100 kHz ^{19}F decoupling was used while recording the ^{13}C spectrum of pristine PTFE. For ^{23}Na NMR measurements a 71.5 kHz RF field amplitude was used and ^{23}Na shifts were referenced to NaF at 7.13 ppm with respect to a 1 M aqueous solution of NaCl at 0 ppm.[2] All spectra were acquired using a Hahn echo (90°-180°) sequence with an inter-pulse delay equal to one rotor period, except ^{23}Na MAS NMR of the solid material recovered by evaporating the aqueous filtrate to dryness, for which a single pulse sequence was used. To make this spectrum as close to quantitative as possible (accounting for the different nutation frequencies of ^{23}Na nuclei in $C_Q = 0$ and $C_Q > 0$ environments; C_Q = quadrupolar coupling constant) a low tip angle (short excitation pulse; 0.2 µs corresponding to ~5°) was used. Measured T_1 values for each species observed and the experimental parameters used to record each spectrum are given in **Supplementary Table S1**.

2. General Procedures

General Procedure A - Upcycling of Fluoride from PTFE Into Sulfonyl Fluorides:

In an argon filled glovebox, sodium metal (69 mg, 3.0 mmol, 4.0 equiv., ~5 mm chunks) and PTFE powder (75 mg, 1.5 mmol, 2.0 equiv. based on a CF_2 repeating unit) were combined in a 13 mL stainless steel ball milling jar with a 10 mm stainless steel ball. The jar was sealed in the glove box under argon. The jar was taken outside the glove box and milled on a Retsch MM400 ball mill at 30 Hz for 60 minutes. After milling the jar was opened in the glove box revealing a black powder. Sulfonyl chloride (0.75 mmol, 1.0 equiv.), tetraethylammonium chloride (248 mg, 1.50 mmol, 2.0 equiv.) and *para*-toluenesulfonic acid (71 mg, 0.38 mmol, 0.5 equiv.) were then added to the jar, which was sealed and milled again at 30 Hz for 60 minutes. The jar was then opened in air and the contents were washed out with ethyl acetate (3 x 10 mL) and filtered through a plug of cotton wool. The solvent was then removed *in vacuo* and the residue was filtered through a plug of basified silica with Et_2O as eluent unless otherwise stated. The solvent was removed *in vacuo* to give the desired sulfonyl fluoride product.

General Procedure B - Upcycling of Fluoride from PTFE Into Acyl Fluorides:

In an argon filled glovebox, sodium metal (69 mg, 3.0 mmol, 4.0 equiv., ~5 mm chunks) and PTFE powder (75 mg, 1.5 mmol, 2.0 equiv. based on a CF_2 repeating unit) were combined in a 13 mL stainless steel ball milling jar with a 10 mm stainless steel ball. The jar was sealed in the glove box under argon. The jar was taken outside the glove box and milled on a Retsch MM400 ball mill at 30 Hz for 60 minutes. After milling the jar was opened in the glove box revealing a black powder. Acyl chloride (0.75 mmol, 1.0 equiv.), tetraethylammonium chloride (248 mg, 1.50 mmol, 2.0 eq.) and pyridinium *p*-toluenesulfonate (189 mg, 0.75 mmol, 1.0 equiv.) were then added to the jar, which was sealed and milled again at 30 Hz for 60 minutes. The jar was then opened in air and the crude reaction mixture was washed out with $CDCl_3$ and filtered through a plug of cotton wool. 1,1,1-Trifluorotoluene (31 µL, 0.25 mmol, 0.33 equiv.) or fluorobenzene (71 µL, 0.75 mmol, 1.0 equiv.) was added to the filtrate and the resulting solution was mixed well and analysed by ^{19}F NMR to determine an NMR yield of the acyl fluoride product.

3. Reductive Defluorination of PTFE

3.1 Solid-state MAS NMR studies

(a) Experimental procedure to obtain sample of crude residue (under argon):

In an argon filled glovebox, sodium metal (69 mg, 3.0 mmol, 2.0 eq., ~5 mm chunks) and PTFE powder (75 mg, 1.5 mmol, 1.0 eq. based on a CF_2 repeating unit) were combined in a Retsch 5 mL stainless steel ball milling jar with an 8 mm stainless steel ball. The jar was sealed in the glove box under argon and wrapped with electrical tape. The jar was taken outside the glove box and milled on a Retsch MM400 ball mill at 30 Hz for 60 minutes. After milling the jar was opened in the glove box revealing a black powder. The powder was packed inside the glove box under argon and was characterised by solid-state MAS NMR (^{19}F, ^{23}Na, ^{13}C).

(b) Experimental procedure to obtain crude sample exposed to air:

In an argon filled glovebox, sodium metal (69 mg, 3.0 mmol, 2.0 eq., ~5 mm chunks) and PTFE powder (75 mg, 1.5 mmol, 1.0 eq. based on a CF_2 repeating unit) were combined in a Retsch 13 mL stainless steel ball milling jar with an 10 mm stainless steel ball. The jar was sealed in the glove box under argon. The jar was taken outside the glove box and milled on a Retsch MM400 ball mill at 30 Hz for 60 minutes. After milling the jar was opened in the glove box revealing a black powder. The jar was left open to air for several days. The resulting solid was characterised by solid-state MAS NMR.

(c) Experimental procedure to obtain washed precipitate and filtrate:

In an argon filled glovebox, sodium metal (207 mg, 9.0 mmol, 2.0 equiv., ~5 mm chunks) and PTFE powder (225 mg, 4.5 mmol, 1.0 eq. based on a CF_2 repeating unit) were combined in a Retsch 5 mL stainless steel ball milling jar with an 8 mm stainless steel ball. The jar was sealed in the glove box under argon and wrapped with electrical tape. The jar was taken outside the glove box and milled on a Retsch MM400 ball mill at 30 Hz for 60 minutes. After milling the jar was opened in air revealing a black powder.

The jar was left open to air for 40 minutes, before deionised water (2 ml) was added to the jar. The mixture was sealed and milled on a MM400 ball mill at 30 Hz for 2 minutes with an 8 mm stainless steel ball. The jar was opened, and the slurry was transferred via pipette to a 15 ml centrifuge tube. The jar was washed with deionised water (7 ml) and added to the centrifuge tube so that the total volume in the centrifuge tube was 9 ml. The mixture was centrifuged at 12000 rpm for 5 minutes.

The colourless liquid was transferred to a separate flask, while deionised water (2 ml) was added to the black solid and the slurry was transferred back to the milling jar. This milling/centrifuging process was conducted another 2 times (3 times in total) in the same manner except that on the final (third) cycle the mixture was centrifuged for 10 minutes instead of 5 minutes.

On the final cycle, once the colourless aqueous washing was removed, leaving the black solid in the centrifuge tube, the black solid was further washed via the addition of deionised water (9 ml) to the centrifuge tube. The mixture was thoroughly mixed and centrifuged at 12000 rpm for 20 minutes. This washing/centrifuging was repeated one further time. On the final centrifuging most of the liquid was removed leaving ~ 2ml in the centrifuge tube, which was further centrifuged for another 5 minutes, however, the mixture could not be further separated.

Deionised water (8 ml) was added to the centrifuge tube (total volume 10 ml) and the mixture was slowly pipetted onto a frit containing filter paper. The black solid collected on top of the filter paper was transferred to a vial and was dried in a vacuum oven set to 80 °C for 30 minutes at <75 mbar, giving a homogeneous black solid (0.0321 g, 59 % yield). The black solid was characterised by solid-state MAS NMR (^{19}F, ^{23}Na and ^{13}C).

The combined aqueous washings from the centrifuging were combined and the solution was filtered via filter paper to remove small carbon impurities. The resulting colourless solution was placed on a rotary evaporator set to 50 °C at 72 mbar for 120 minutes, which gave a while solid. The solid was further dried by connecting the flask to a Schlenk line and placed under dynamic vacuum for 3 hours, with an oil bath set to 150 °C, giving NaF (0.3479 g, 92% yield), which was characterised by solid-state NMR (^{19}F, ^{23}Na) matching a commercial sample of NaF.

(d) T_1 measurements and experimental parameters used to record solid-state MAS NMR

**Sample**	**Nucleus**	**Figure**	**Pulse sequence**	**T_1 (s)**	**Pulse length (μs)**	**Recycle delay (s)**	**Number of scans**	**Experiment time (h)**
PTFE	^{13}C	2d	echo	*n/d*	3.5 (90°)	5	160	0.22
	^{19}F	2b	echo	*n/d*	2.5 (90°)	10	76	0.21
NaF	^{19}F	2b	echo	*n/d*	2.5 (90°)	10	16	0.04
	^{23}Na	2c	echo	*n/d*	3.5 (90°, C_Q ~ 0)	10	16	0.04
Dried aqueous filtrate	^{19}F	2b	echo	NaF: 0.95	2.5 (90°)	10	48	0.13
	^{23}Na	2c	Bloch decay, low tip angle	NaF: 8.0	0.2 (~5°)	40	40	0.44
	^{23}Na, T_1 filter	2c	Bloch decay, low tip angle			0.05	1662	0.02
Crude (air free)	^{13}C		echo	*n/d*	3.5 (90°)	5	27112	37.66
	^{19}F	S1	echo	NaF: 0.63 PTFE: 0.43	2.5 (90°)	5	68	0.09
	^{23}Na	S5	echo	*n/d*	3.5 (90°, C_Q ~ 0)	5	20	0.03
Crude (air exposed)	^{13}C	S4	echo	*n/d*	3.5 (90°)	5	14416	20.02
Precipitate (after washing)	^{13}C	2d	echo	*n/d*	3.5 (90°)	5	47012	65.29
	^{19}F	S1	echo	*n/d*	2.5 (90°)	5	112	0.16
	^{23}Na	S5	echo	*n/d*	3.5 (90°, C_Q ~ 0)	5	68	0.09

Supplementary Table S1: Experimentally measured spin-lattice relaxation times (T_1) and acquisition parameters used to record MAS NMR spectra.

e) Data obtained from solid-state MAS NMR:

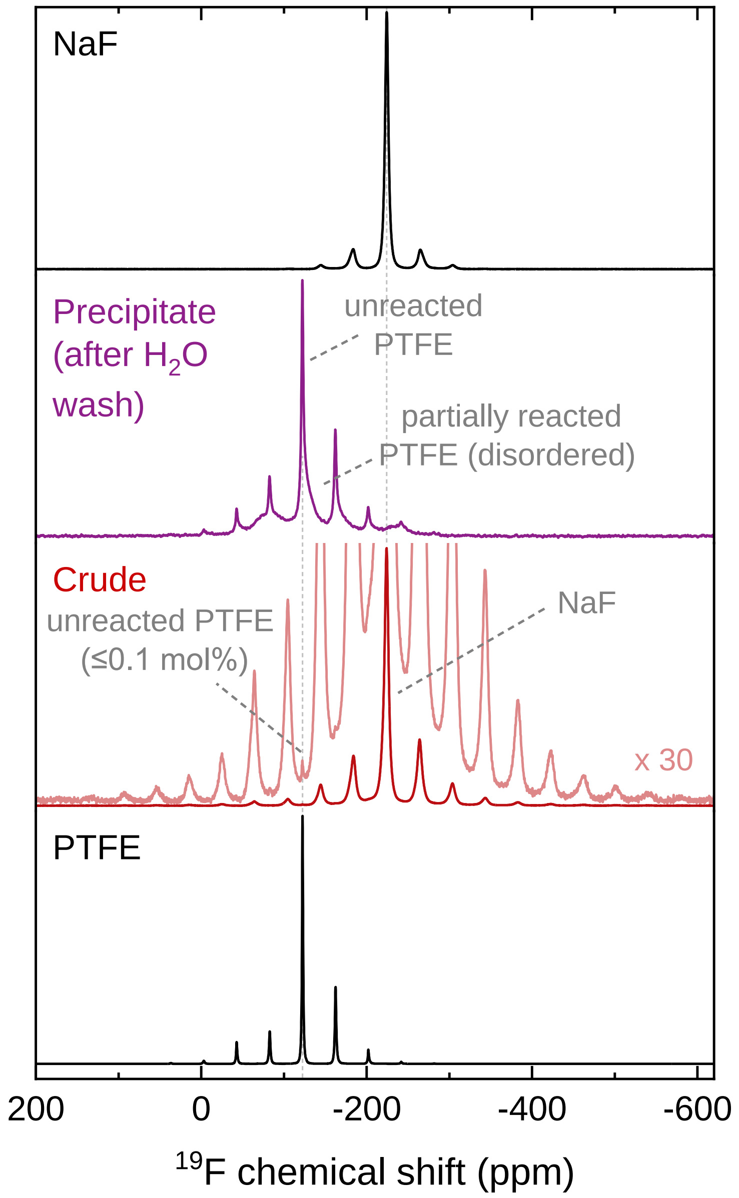

Supplementary Figure S1: ^{19}F MAS NMR analysis of crude residue from mechanochemical defluorination, which has not been exposed to air, and of the black precipitate recovered by filtration after washing this crude material repeatedly with water. ^{19}F spectra of pristine NaF and PTFE are shown for comparison. Echo-detected, 15 kHz MAS, 9.4 T, RT. For acquisition parameters, see Table S1.

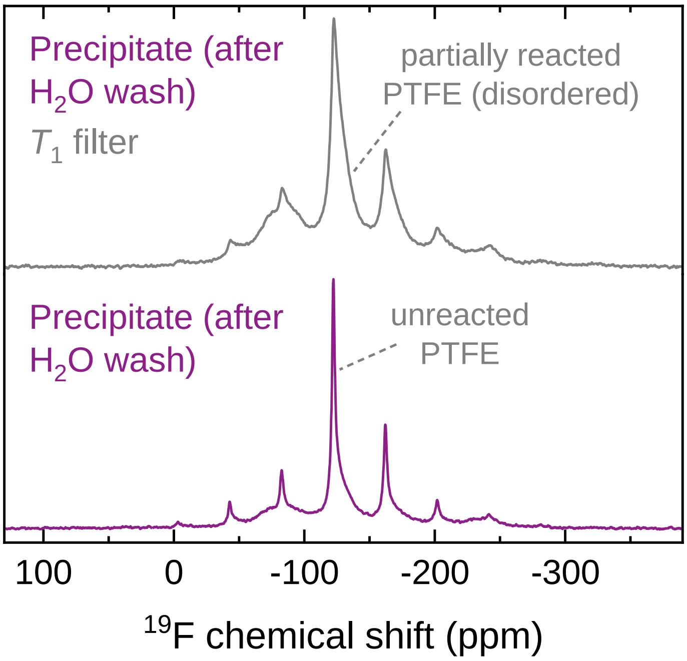

Supplementary Figure S2: ^{19}F MAS NMR analysis of black precipitate recovered by filtration after washing the crude residue repeatedly with water. The quantitative ^{19}F spectrum is shown (bottom; recycle delay, 5s) and compared to a spectrum in which a T_1 filter has been applied (recycle delay, 0.1s). Use of a shorter recycle delay leads to a spectrum in which species with rapid spin-lattice relations (short T_1) are over-represented. By this method we are able to identify a minority second ^{19}F environment in the precipitate, likely corresponding to a very small quantity of partially reacted and thus highly disordered PTFE. Static disorder and defects in materials typically lead to NMR signal broadening and short T_1 values. Echo-detected, 15 kHz MAS, 9.4 T, RT. For acquisition parameters, see Table S1.

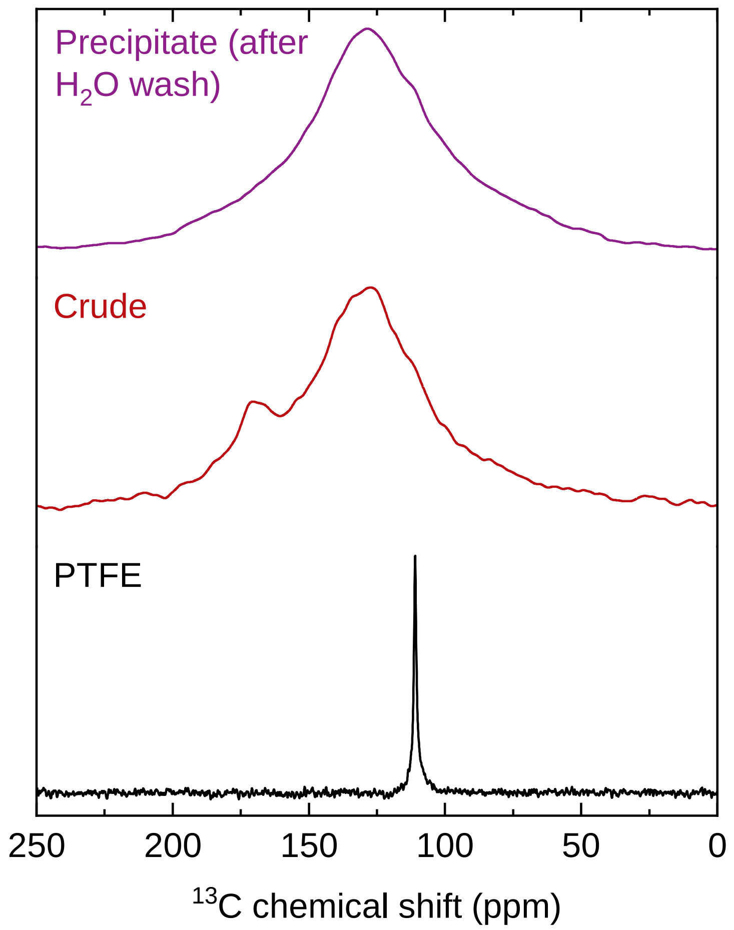

Supplementary Figure S3: ^{13}C MAS NMR analysis of crude residue from mechanochemical defluorination and of the black precipitate recovered by filtration after washing this crude material repeatedly with water. The ^{13}C spectrum of pristine PTFE is shown for comparison. In the ^{13}C spectrum of the crude residue, a second broad ^{13}C signal is observed, centred at δ_C = 168.9 ppm. This signal is reproducibly present in the crude material, regardless of whether this material has been exposed to ambient air or measured under argon (see **Supplementary Figure S4**). The identity of the ^{13}C environment(s) giving rise to this signal are unknown. However, we speculate that they may correspond to an sp^2 carbon environment involved in coordination with fluoride ions in the crude residue, which are subsequently removed during washing. Echo-detected, 15 kHz MAS, 9.4 T, RT. For acquisition parameters, see Table S1.

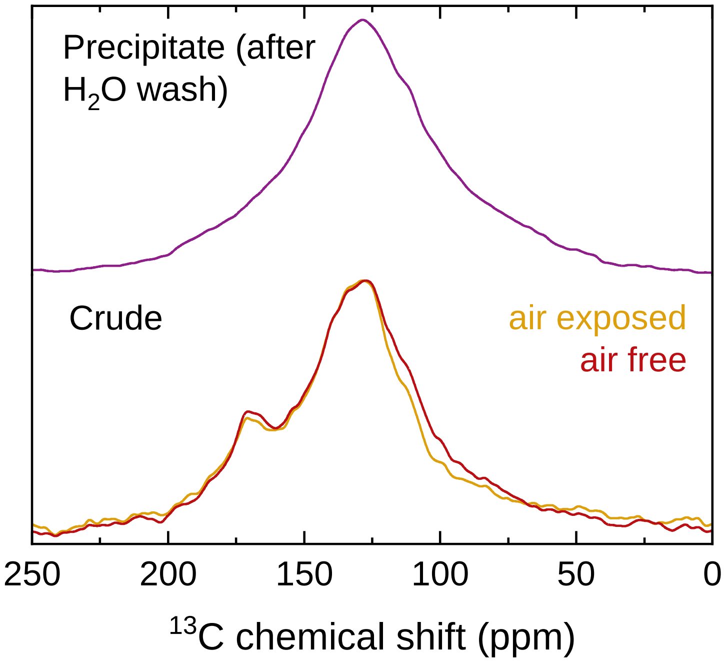

Supplementary Figure S4: ^{13}C MAS NMR analysis of crude residue from mechanochemical defluorination (bottom) and of the black precipitate recovered by filtration after washing this crude material repeatedly with water (top). ^{13}C spectra recorded when the crude residue is (orange) and is not (red) exposed to ambient air prior to NMR analysis are shown. Echo-detected, 15 kHz MAS, 9.4 T, RT. For acquisition parameters, see Table S1.

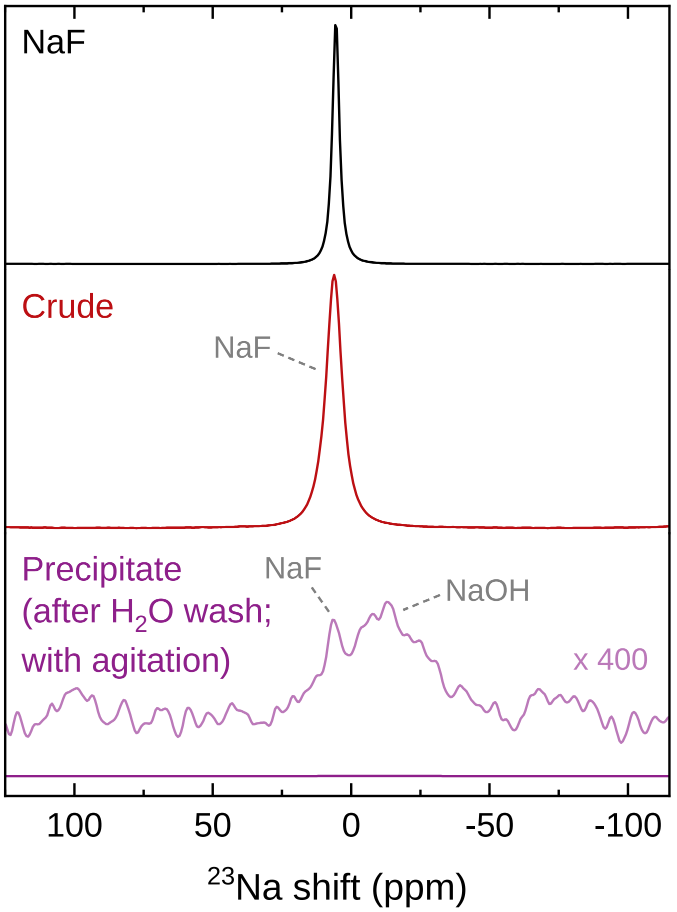

Supplementary Figure S5: ^{23}Na MAS NMR analysis of the crude residue from mechanochemical defluorination and of the black precipitate recovered by filtration after washing this crude material repeatedly with agitation in a mixer mill. The ^{23}Na spectrum of pristine NaF is shown for comparison. The spectra are scaled by the number of transients recorded such as to show the approximate relative quantities of Na found in each sample. Echo-detected, 15 kHz MAS, 9.4 T, RT. For acquisition parameters, see Table S1.

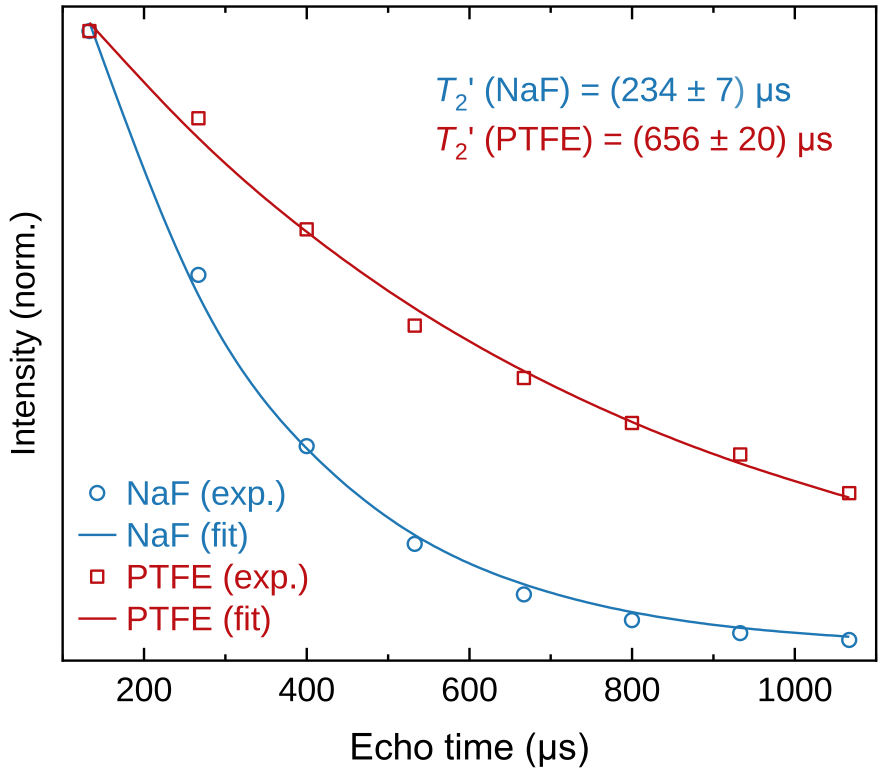

Supplementary Figure S6: ^{19}F MAS NMR variable echo delay T_2 measurement of crude residue from mechanochemical defluorination. Spectra were recorded with increasing, rotor-synchronised, echo delay time to investigate differences in the spin-spin relaxation times (T_2) of NaF and unreacted PTFE.

*(f) **Supplementary Note 1** - the effect of T_2 on quantification*

When conducting quantitative ^{19}F MAS NMR experiments, we were careful to first confirm if any differences in ^{19}F T_2 values of NaF and PTFE are present that could lead to T_2-weighting and skewed quantification. We found that the unreacted PTFE has a slightly longer T_2 (0.66 ms) compared to NaF (0.23 ms), and thus – if any T_2 filtering does occur in the quantitative ^{19}F MAS NMR spectrum shown in Scheme 2b – the small quantity of unreacted PTFE is overrepresented, and we do not inadvertently overestimate the conversion efficacy of our method. Since there might be a discernible effect of T_2 decoherence on the amount of signal in the echo, we evaluate it as follows:

rotor period, τ = 66.67 μs; echo time, 2τ = 133.34 μs; to calculate the fraction remaining in the echo, we scale the PTFE and NaF integrals by their relative scaling factors: $\exp(-2\tau/T_2)$.

PTFE: T_2 = (0.66 ± 0.02) ms, fraction remaining: exp(-0.133/0.66) = 0.817; i.e. 82% remained, 17% decayed

NaF: T_2 = (0.234 ± 0.007) ms, fraction remaining: exp(-0.133/0.234) = 0.561; i.e. 56% remained, 44% decayed

There is indeed a discernible effect of T_2 decoherence on the amount of signal in the echo. To understand its importance quantitatively, we use the following approach. We consider two components with transverse relaxation times $T_{2,\mathrm{L}}$ (long T_2 - PTFE) and $T_{2,\mathrm{S}}$ (short T_2 - NaF). Let F denote the true (zero-time, t = 0) fraction of the long T_2 component, and O the observed fraction measured at an echo time t. The observed fraction is T_2-weighted, and can be written as

$$O = \frac{F\, e^{-t/T_{2,L}}}{F\, e^{-t/T_{2,L}} + (1-F)\, e^{-t/T_{2,S}}}$$

We define the relative attenuation factor as

$$r \equiv \frac{e^{-t/T_{2,L}}}{e^{-t/T_{2,S}}} = e^{-t\left(\frac{1}{T_{2,L}} - \frac{1}{T_{2,S}}\right)}$$

leading to the following compact expression for the observed fraction:

$$O = \frac{F\, r}{1 + F\,(r-1)}$$

The absolute bias can then be expressed as

$$\Delta \equiv O - F = \frac{F(1-F)\,(r-1)}{1 + F\,(r-1)}$$

and for small F (i.e. small amount of the long T_2 component, PTFE, F≪1), approximately as

$$\Delta \approx F\,(r-1)$$

In our case, taking $T_{2,L}$ = (0.66 ± 0.02) ms for PTFE and T_2 = (0.234 ± 0.007) ms for NaF, we obtain r = 1.459. So, for example, if the true fraction of PTFE is F = 0.1% and Δ = 0.459*F = 0.0459%, we obtain an observed fraction of O = Δ + F = 0.1459% which is 0.0459 percent point higher than the true fraction of 0.1%, a very small difference indeed. We therefore conclude that the effect of T_2 filtering is negligible. In practice, we chose to conservatively give the approximate fraction of PTFE as ≤0.1 mol% because the signal of PTFE is so small that its integral, in the presence of the substantially more intense signal of NaF, needs to be interpreted with caution.

3.2 Solution state ^{19}F NMR studies:

In an argon filled glovebox, sodium metal (69 mg, 3.0 mmol, 4.0 eq., ~5 mm chunks) and PTFE powder (75 mg, 1.5 mmol, 2.0 eq. based on a CF_2 repeating unit) were combined in a 13 mL stainless steel ball milling jar with a 10 mm stainless steel ball. The jar was sealed in the glove box under argon. The jar was taken outside the glove box and milled on a Retsch MM400 ball mill at 30 Hz for 60 minutes. After milling the jar was opened in air revealing a black powder.

The jar was left open to air for 40 minutes, before deionised water (2 ml) was added to the jar. The mixture was sealed and milled on a MM400 ball mill at 30 Hz for 2 minutes with a 10 mm stainless steel ball. The jar was opened, and the slurry was transferred via pipette to a 15 ml centrifuge tube. The jar was washed with deionised water (7 ml) and added to the centrifuge tube so that the total volume in the centrifuge tube was 9 ml. The mixture was centrifuged at 6500 rpm for 20 minutes.

The colourless liquid was transferred to a separate flask, while deionised water (2 ml) was added to the black solid and the slurry was transferred back to the milling jar. This milling/centrifuging process was conducted another 2 times (3 times in total). Hexafluoro-2-propanol (53 µL, 0.5 mmol, 0.17 eq.) was added to the combined colourless supernatants and 0.9 mL of this solution was added to an NMR tube, along with 0.1 mL of D_2O.

The quantitative ^{19}F NMR spectrum is shown below. Based upon this data, the NMR yield of NaF was determined to be 98%.

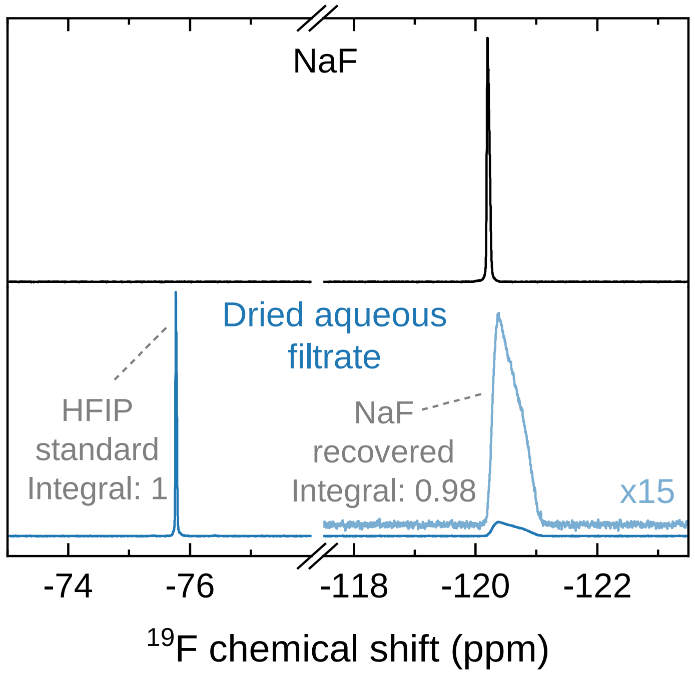

Supplementary Figure S7: ^{19}F solution-NMR spectrum (282 MHz, H_2O/D_2O 9:1, 298 K) of crude extracted NaF along with 0.17 equivalents of hexafluoro-2-propanol (HFIP). Note that the fluoride signal shows a broad line-shape in the quantitative spectrum. A broad fluoride signal was also observed when a mixture of HFIP and pristine NaF was analysed under identical conditions, and accordingly, we tentatively attribute this broadening to an interaction between NaF and HFIP.

3.3 Temperature studies:

In an argon filled glovebox, sodium metal (69 mg, 3.0 mmol, 4.0 equiv., ~5 mm chunks) and PTFE powder (75 mg, 1.5 mmol, 2.0 equiv. based on a CF_2 repeating unit) were combined in a 13 mL stainless steel ball milling jar with a 10 mm stainless steel ball. The jar was sealed in the glove box under argon. The jar was taken outside the glove box and milled on a Retsch MM400 ball mill at 30 Hz for 60 minutes and the external temperature of the jar was recorded by thermocouple at intervals. A control jar containing only PTFE (75 mg) and one 10 mm diameter stainless steel ball was also milled at 30 Hz for 60 mins and the temperature recorded at the same intervals.

**Time (mins)**	**Temperature of Reaction Jar (°C)**	**Temperature of Control Jar (°C)**
0	27.9	27.4
1	27.0	27.0
2	27.6	27.0
5	27.5	26.5
10	27.8	26.0
20	27.8	25.4
30	27.4	25.6
40	27.1	25.9
50	26.9	26.2
60	26.6	26.6

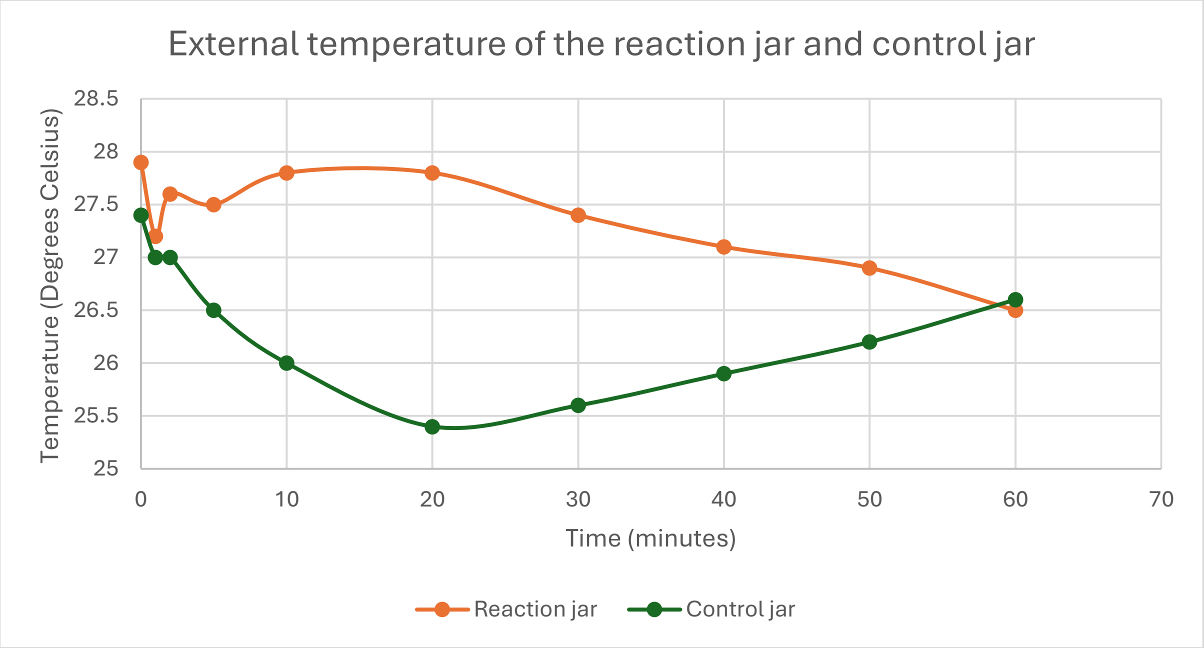

3.4 Source of PTFE

To explore the reproducibility of our method, we tested Na-mediated degradation of PTFE from several different suppliers, including both powder and tape, according to the following protocol:

In an argon filled glovebox, sodium metal (69 mg, 3.0 mmol, 4.0 eq., ~5 mm chunks) and PTFE (75 mg, 1.5 mmol, 2.0 eq. based on a CF_2 repeating unit) were combined in a 13 mL stainless steel ball milling jar with a 10 mm stainless steel ball. The jar was sealed in the glove box under argon. The jar was taken outside the glove box and milled on a Retsch MM400 ball mill at 30 Hz for 60 minutes. After milling the jar was opened in air revealing a black powder.

The jar was left open to air for 40 minutes, before deionised water (2 ml) was added to the jar. The mixture was sealed and milled on a MM400 ball mill at 30 Hz for 2 minutes with a 10 mm stainless steel ball. The jar was opened, and the slurry was transferred via pipette to a 15 ml centrifuge tube. The jar was washed with deionised water (7 ml) and added to the centrifuge tube so that the total volume in the centrifuge tube was 9 ml. The mixture was centrifuged at 6500 rpm for 20 minutes.

The colourless liquid was transferred to a separate flask, while deionised water (2 ml) was added to the black solid and the slurry was transferred back to the milling jar. This milling/centrifuging process was conducted another 2 times (3 times in total). Hexafluoro-2-propanol (53 µL, 0.5 mmol, 0.17 eq.) was added to the combined colourless supernatants and 0.9 mL of this solution was added to an NMR tube, along with 0.1 mL of D_2O, based upon which, the ^{19}F NMR yield of NaF was determined.

The results from these experiments are shown in **Supplementary Table S2**

**Supplier**	**Catalogue Number**	**Form**	**Specifications**	**Yield NaF (%)[a]**
Thermo Fisher Scientific	178930050	Powder	Not available	98
Sigma Aldrich	430935-5G	Powder	1 µm particle size	98
Amazon	B08P58HCSS	Powder	"Pure, 100% Polytetrafluoroethylene dry lubricant, 2-3µm fine powder"	99
Fischer Scientific	15698250 (VITLAB™ 131097)	Tape	Width 12 mm, torn into ~10 mm lengths.	98
RS Components	196-9893	Rod	25 mm Diameter rod cut into small pieces (~5 x 1 x 1 mm)	99

Supplementary Table S2: Results of defluorination of PTFE purchased from different suppliers. [a] Yields of NaF determined by quantitative solution-state ^{19}F NMR analysis with hexafluoro-2-propanol as an internal standard.

4. Upcycling of Fluoride from PTFE into Fine Chemicals

4.1 Optimisation of reaction conditions:

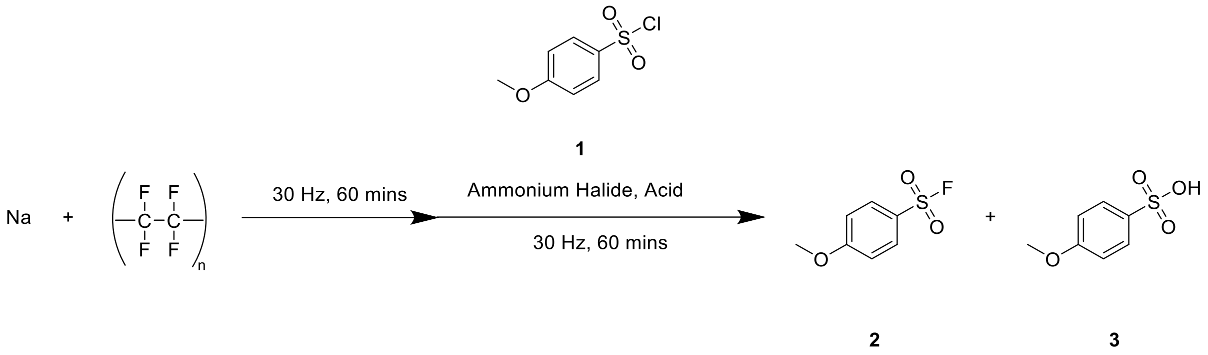

In an argon filled glovebox, sodium metal (69 mg, 3.0 mmol, 4.0 equiv., ~5 mm chunks) and PTFE powder (75 mg, 1.5 mmol, 2.0 equiv. based on a CF_2 repeating unit) were combined in a 13 mL stainless steel ball milling jar with a 10 mm stainless steel ball. The jar was sealed in the glove box under argon. The jar was taken outside the glove box and milled on a Retsch MM400 ball mill at 30 Hz for 60 minutes. After milling the jar was opened in the glove box revealing a black powder. 4-methoxybenzenesulfonyl chloride (155 mg, 0.75 mmol, 1.0 eq.), ammonium salt and acid were then added to the jar, which was sealed and milled again at 30 Hz for 60 minutes. The jar was then opened in air and the crude reaction mixture was washed out with $CDCl_3$ and filtered through a plug of cotton wool. 1,1,1-Trifluorotoluene (31 µL, 0.25 mmol, 0.33 equiv.) and 1,1,2,2-tetrachloroethane (79 µL, 0.38 mmol, 0.5 eq.) were added to the filtrate and the resulting solution was mixed well and analysed by ^{19}F NMR to determine an NMR yield of the acyl fluoride product 2 and by ^{1}H NMR to determine NMR yields of remaining sulfonyl chloride **1** and sulfonic acid **3**.

**Entry**	**Acid (eq.)**	**Ammonium Salt (eq.)**	**^{1}H NMR Yield of 1**	**^{19}F NMR Yield of 2**	**^{1}H NMR Yield of 3**
1	None	None	55%	<5%	<5%
2	None	Et_4NCl (1.5)	24%	13%	<5%
3	Citric Acid (0.3)	Et_4NCl (1.5)	10%	67%	11%
4	PyHCl (0.3)	Et_4NCl (1.5)	<5%	34%	<5%
5	Tartaric Acid (0.3)	Et_4NCl (1.5)	32%	54%	14%
6	Benzoic Acid (0.3)	Et_4NCl (1.5)	<5%	81%	17%
7	CSA (0.3)	Et_4NCl (1.5)	<5%	43%	12%
8	PTSA (0.3)	Et_4NCl (1.5)	<5%	90%	<5%
9	PTSA (0.5)	Et_4NCl (1.5)	N.D.	96%	<5%

10	PTSA (0.5)	Bu_4NCl (1.5)	69%	4%	20%
11	PTSA (0.5)	Me_4NCl (1.5)	46%	9%	<5%
12	PTSA (0.5)	Et_4NBr (1.5)	18%	7%	<5%
13	PTSA (0.5)	Et_4NI (1.5)	12%	15%	<5%
14	PTSA (0.5)	Et_4NOTs (1.5)	<5%	69%	<5%
15	PTSA (0.5)	Et_4NCl (2.0)	N.D.	Quant	<5%
16	PTSA (0.5)	None	90%	<5%	<5%

4.2 Experimental procedures:

4-Methoxybenzenesulfonyl fluoride, **1a**

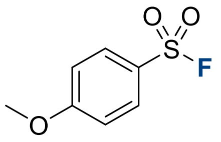

4-Methoxybenzenesulfonyl chloride (155 mg, 0.75 mmol, 1.0 eq.) was subjected to general procedure A to give the title compound as a white crystalline solid (136 mg, 95%).

^{1}H NMR (300 MHz, CDCl$_3$) δ$_H$: 7.96 – 7.89 (m, 2H), 7.09 – 7.02 (m, 2H), 3.91 (s, 3H).

^{13}C NMR (75 MHz, CDCl$_3$) δ$_C$: 165.4, 130.9, 124.1 (d, *J* = 24.5 Hz), 115.0, 56.0.

^{19}F NMR (282 MHz, CDCl$_3$) δ$_F$: 67.3 (s).

All data are consistent with literature values.[3]

4-Methylbenzenesulfonyl fluoride, **1b**

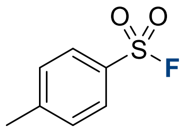

4-Methylbenzenesulfonyl chloride (143 mg, 0.75 mmol, 1.0 eq.) was subjected to general procedure A to give the title compound as a white crystalline solid (119 mg, 91%).

^{1}H NMR (400 MHz, CDCl$_3$) δ$_H$: 7.91 – 7.87 (m, 2H), 7.42 (d, *J* = 8.1 Hz, 2H), 2.49 (s, 3H).

^{13}C NMR (101 MHz, CDCl$_3$) δ$_C$: 147.2, 130.2 (d, *J* = 26.3 Hz), 130.1, 128.6, 22.0.

^{19}F NMR (282 MHz, CDCl$_3$) δ$_F$: 66.3 (s).

All data are consistent with literature values.[3]

(1,1'-Biphenyl)-4-sulfonyl fluoride, **1c**

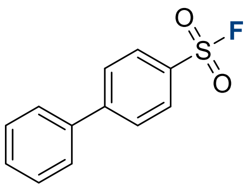

(1,1'-Biphenyl)-4-sulfonyl fluoride chloride (190 mg, 0.75 mmol, 1.0 eq.) was subjected to general procedure A to give the title compound as a white crystalline solid (139 mg, 79%).

^{1}H NMR (300 MHz, CDCl$_3$) δ$_H$: 8.13 – 8.03 (m, 2H), 7.87 – 7.78 (m, 2H), 7.67 – 7.59 (m, 2H), 7.58 – 7.45 (m, 3H).

^{13}C NMR (101 MHz, CDCl$_3$) δ$_C$: 148.8, 138.7, 131.5 (d, J = 24.6 Hz), 129.4, 129.3, 129.1, 128.3, 127.6.

^{19}F NMR (282 MHz, CDCl$_3$) **δ$_F$:** 66.5 (s)

All data are consistent with literature values.[4]

4-Acetamidobenzenesulfonyl fluoride, **1d**

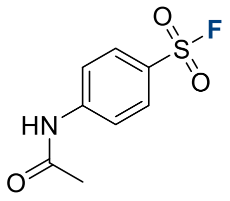

4-Acetamidobenzenesulfonyl chloride (175 mg, 0.75 mmol, 1.0 eq.) was subjected to general procedure A to give the title compound as a white crystalline solid (146 mg, 90%).

^{1}H NMR (300 MHz, CDCl$_3$) δ$_H$: 8.00 – 7.92 (m, 2H), 7.83 – 7.74 (m, 2H), 7.53 (s, 1H), 2.25 (s, 3H).

^{13}C NMR (75 MHz, CDCl$_3$) δ$_C$: 168.8, 144.6, 130.2, 127.3 (d, J = 25.0 Hz), 119.5, 25.0.

^{19}F NMR (376 MHz, CDCl$_3$) **δ$_F$:** 66.8 (s)

All data are consistent with literature values.[5]

Naphthalene-2-sulfonyl fluoride, **1e**

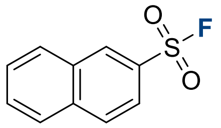

Naphthalene-2-sulfonyl chloride (170 mg, 0.75 mmol, 1.0 eq.) was subjected to general procedure A to give the title compound as a white crystalline solid (122 mg, 77%).

^{1}H NMR (400 MHz, CDCl$_3$) δ$_H$: 8.68 – 8.56 (m, 1H), 8.10 – 8.00 (m, 2H), 7.96 (m, 2H), 7.73 (m, 2H).

^{13}C NMR (101 MHz, CDCl$_3$) δ$_C$: 136.2, 132.0, 131.1, 130.5, 130.2, 130.0 (d, J = 24.6 Hz), 129.8, 128.5, 128.3, 122.3.

^{19}F NMR (282 MHz, CDCl$_3$) **δ$_F$:** 66.3 (s)

All data are consistent with literature values.[6]

4-(Trifluoromethyl)benzenesulfonyl fluoride, **1f**

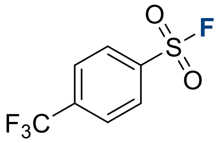

4-(Trifluoromethyl)benzenesulfonyl chloride (184 mg, 0.75 mmol, 1.0 eq.) was subjected to general procedure A to give the title compound as a white crystalline solid (81 mg, 48%).

^{1}H NMR (400 MHz, $CDCl_3$) δ_H: 8.17 (d, J = 8.3 Hz, 2H), 7.92 (d, J = 8.3 Hz, 2H).

^{13}C NMR (101 MHz, $CDCl_3$) δ_C: 137.4 (q, J = 33.4 Hz), 136.7 (d, J = 26.6 Hz), 129.3, 127.1 (q, J = 3.7 Hz), 122.9 (q, J = 273.2 Hz).

^{19}F NMR (376 MHz, $CDCl_3$) δ_F: 65.9 (s, 1F), –63.5 (s, 3F).

All data are consistent with literature values.[7]

2-Nitrobenzenesulfonyl fluoride, **1g**

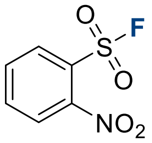

2-Nitrobenzenesulfonyl chloride (166 mg, 0.75 mmol, 1.0 eq.) was subjected to general procedure A to give the title compound as a white crystalline solid (50 mg, 33%).

^{1}H NMR (300 MHz, $CDCl_3$) δ_H: 8.26 (dd, J = 7.8, 1.6 Hz, 1H), 8.05 (dt, J = 7.9, 1.0 Hz, 1H), 7.96 (td, J = 7.7, 1.5 Hz, 1H), 7.88 (tt, J = 7.6, 1.3 Hz, 1H).

^{13}C NMR (75 MHz, $CDCl_3$) δ_C: 136.7, 133.4, 132.0 (d, J = 1.8 Hz), 127.3 (d, J = 28.8 Hz), 126.0.

^{19}F NMR (282 MHz, $CDCl_3$) δ_F: 65.1 (s).

All data are consistent with literature values, except for an absent quaternary ^{13}C NMR signal at 148.3 ppm (N.B. this signal shows low intensity in the literature data).[6]

Butane-1-sulfonyl fluoride, **1h**

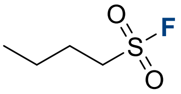

Butane-1-sulfonyl chloride (105 mg, 0.75 mmol, 1.0 eq.) was subjected to a modification of general procedure A, whereby after the mechanochemical fluorination reaction the crude reaction mixture was washed out with $CDCl_3$ and filtered through a plug of cotton wool. 1,1,1-Trifluorotoluene (31 μL, 0.25 mmol, 0.33 equiv.) was added to the filtrate and the

resulting solution was mixed well and analysed by ^{19}F NMR to determine an NMR yield of the acyl fluoride product. The ^{19}F NMR yield = 48%

^{19}F NMR (282 MHz, CDCl$_3$) δ$_F$: 53.3 (t, J = 4.2 Hz).

All data are consistent with literature values.[8]

((1*S*,4*R*)-7,7-Dimethyl-2-oxobicyclo[2.2.1]heptan-1-yl)methanesulfonyl fluoride, **1i**

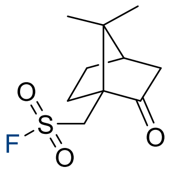

((1*S*,4*R*)-7,7-Dimethyl-2-oxobicyclo[2.2.1]heptan-1-yl)methanesulfonyl chloride (188 mg, 0.75 mmol, 1.0 eq.) was subjected to general procedure A to give the title compound as a white crystalline solid (94 mg, 54%).

^{1}H NMR (400 MHz, CDCl$_3$) δ$_H$: 3.86 (dd, *J* = 15.2, 2.6 Hz, 1H), 3.29 (dd, *J* = 15.2, 2.9 Hz, 1H), 2.51 – 2.28 (m, 2H), 2.20 – 1.95 (m, 3H), 1.74 (ddd, *J* = 13.7, 9.4, 4.5 Hz, 1H), 1.49 (ddd, *J* = 13.0, 9.3, 3.8 Hz, 1H), 1.13 (s, 3H), 0.92 (s, 3H).

^{13}C NMR (101 MHz, CDCl$_3$) δ$_C$: 213.2, 58.0, 48.5 (d, *J* = 18.1 Hz), 48.2, 43.1, 42.5, 27.0, 25.3, 19.8, 19.8.

^{19}F NMR (376 MHz, CDCl$_3$) δ$_F$: 64.2 (t, *J* = 3.1 Hz).

All data are consistent with literature values.[9]

Phenylmethanesulfonyl fluoride, 1j

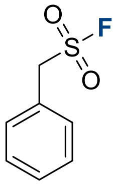

Phenylmethanesulfonyl chloride (143 mg, 0.75 mmol, 1.0 eq.) was subjected to general procedure A to give the title compound as a white crystalline solid (63 mg, 48%).

^{1}H NMR (300 MHz, CDCl$_3$) δ$_H$: 7.50 – 7.39 (m, 5H), 4.60 (d, *J* = 3.2 Hz, 2H).

^{13}C NMR (101 MHz, CDCl$_3$) δ$_C$: 130.8 (d, *J* = 1.1 Hz), 130.1, 129.5, 125.6, 57.0 (d, *J* = 17.8 Hz).

^{19}F NMR (282 MHz, CDCl$_3$) δ$_F$: 51.4 (t, J = 3.2 Hz).

All data are consistent with literature values.[6]

4-Methoxybenzoyl fluoride, **1k**

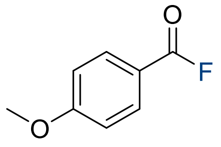

4-Methoxybenzoyl chloride (128 mg, 0.75 mmol, 1.0 eq.) was subjected to a modification of general procedure B, whereby after the mechanochemical fluorination reaction the crude reaction mixture was washed from the milling jar with EtOAc (3 x 10 mL) and filtered through a plug of cotton wool. The solvent was removed in vacuo and the residue was purified by column chromatography through basified silica (petroleum ether: EtOAc, 98:2) to give the title compound as a colourless oil (96 mg, 83%).

^{1}H NMR (400 MHz, CDCl$_3$) δ$_H$: 8.03 – 7.94 (m, 2H), 7.02 – 6.94 (m, 2H), 3.89 (s, 3H).

^{13}C NMR (101 MHz, CDCl$_3$) δ$_C$: 165.4, 157.4 (d, J = 340.0 Hz)., 133.9 (d, J = 4.2 Hz), 117.0 (d, J = 61.8 Hz), 114.6, 55.8.

^{19}F NMR (376 MHz, CDCl$_3$) δ$_F$: 15.9 (s)

All data are consistent with literature values.[10]

4-Methylbenzoyl fluoride, **1l**

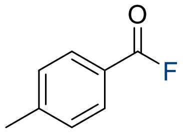

4-Methylbenzoyl chloride (116 mg, 0.75 mmol, 1.0 eq.) was subjected to general procedure B. The yield of the title compound was determined by the addition of 1,1,1-triflorotoluene (31 µL, 0.25 mmol, 0.33 eq.) as an internal standard. The ^{19}F NMR yield = 79%.

^{19}F NMR (282 MHz, CDCl$_3$) δ$_F$: 17.4 (s)

All data are consistent with literature values.[11]

4-(Trifluoromethyl)benzoyl fluoride, **1m**

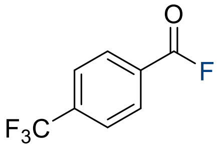

4-(Trifluoromethyl)benzoyl chloride (171 mg, 0.75 mmol, 1.0 eq.) was subjected to general procedure B. The yield of the title compound was determined by the addition of fluorobenzene (71 µL, 0.75 mmol, 1.00 eq.) as an internal standard. The ^{19}F NMR yield = 94%.

^{19}F NMR (282 MHz, CDCl$_3$) δ_F: 20.0 (s, 1F), –63.5 (s, 3F)

All data are consistent with literature values.[12]

Cinnamoyl fluoride, **1n**

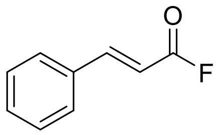

Cinnamoyl chloride (125 mg, 0.75 mmol, 1.0 eq.) was subjected to general procedure C. The yield of the title compound was determined by the addition of 1,1,1-trifluorotoluene (31 µL, 0.25 mmol, 0.33 eq.) as an internal standard. The ^{19}F NMR yield = 73%.

^{19}F NMR (282 MHz, CDCl$_3$) δ_F: 25.5 (d, J = 7.4 Hz).

All data are consistent with literature values.[13]

4.3 NMR spectra:

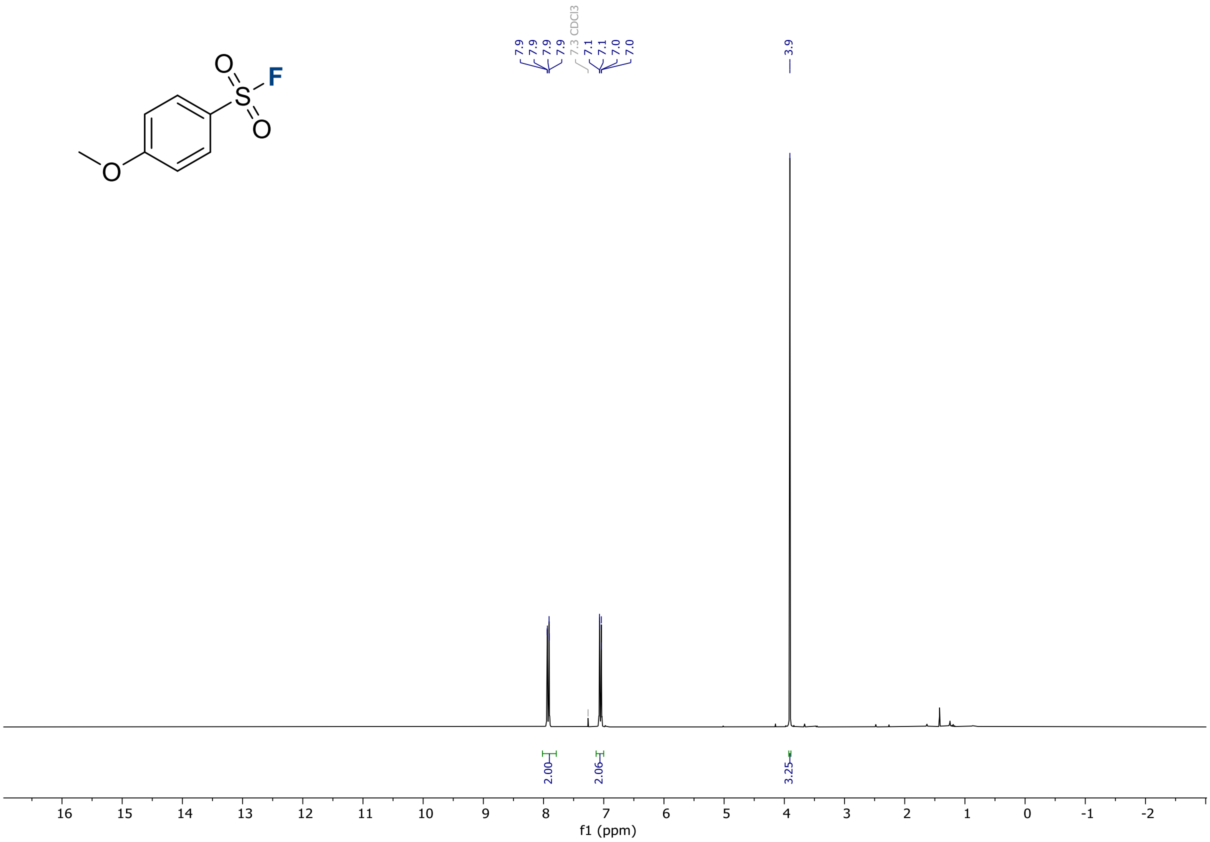

^{1}H NMR (300 MHz, $CDCl_3$, 298 K) spectrum of **1a**.

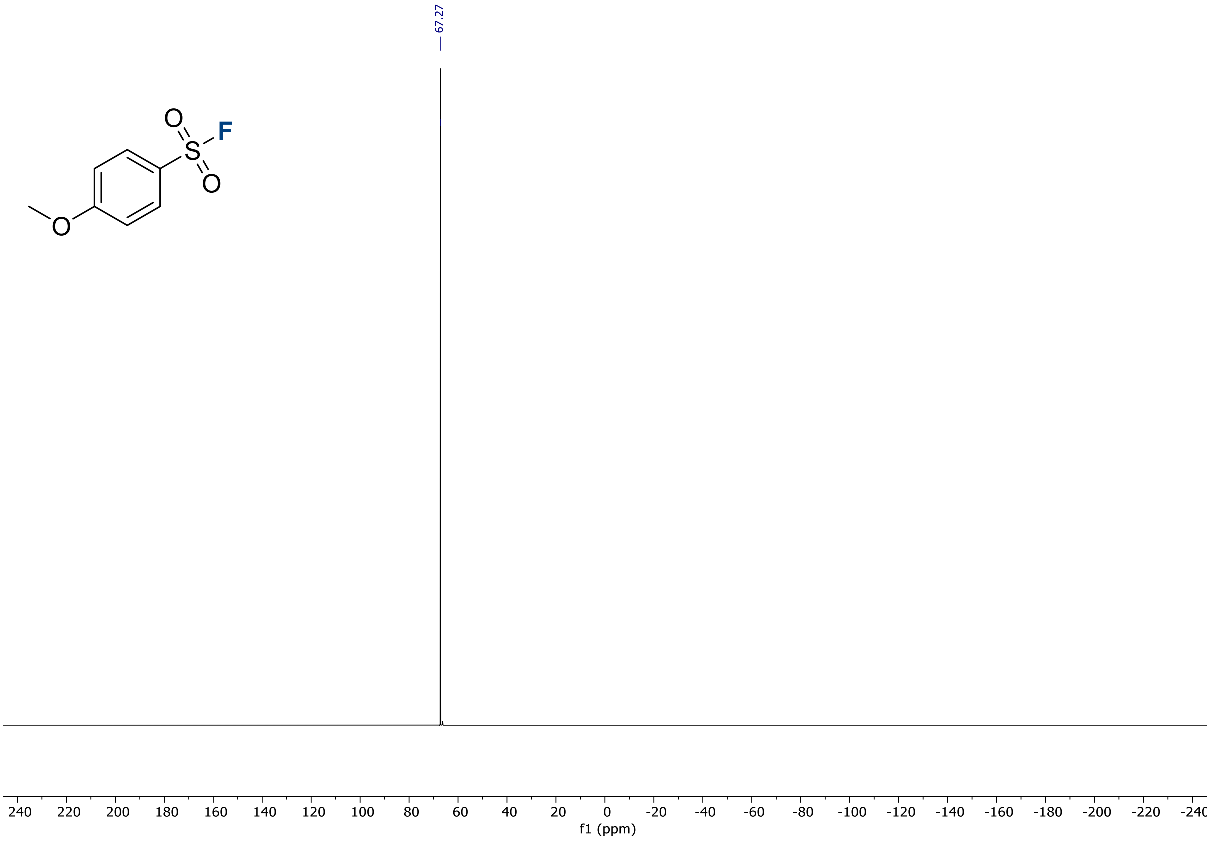

^{19}F NMR (282 MHz, $CDCl_3$, 298 K) spectrum of **1a**.

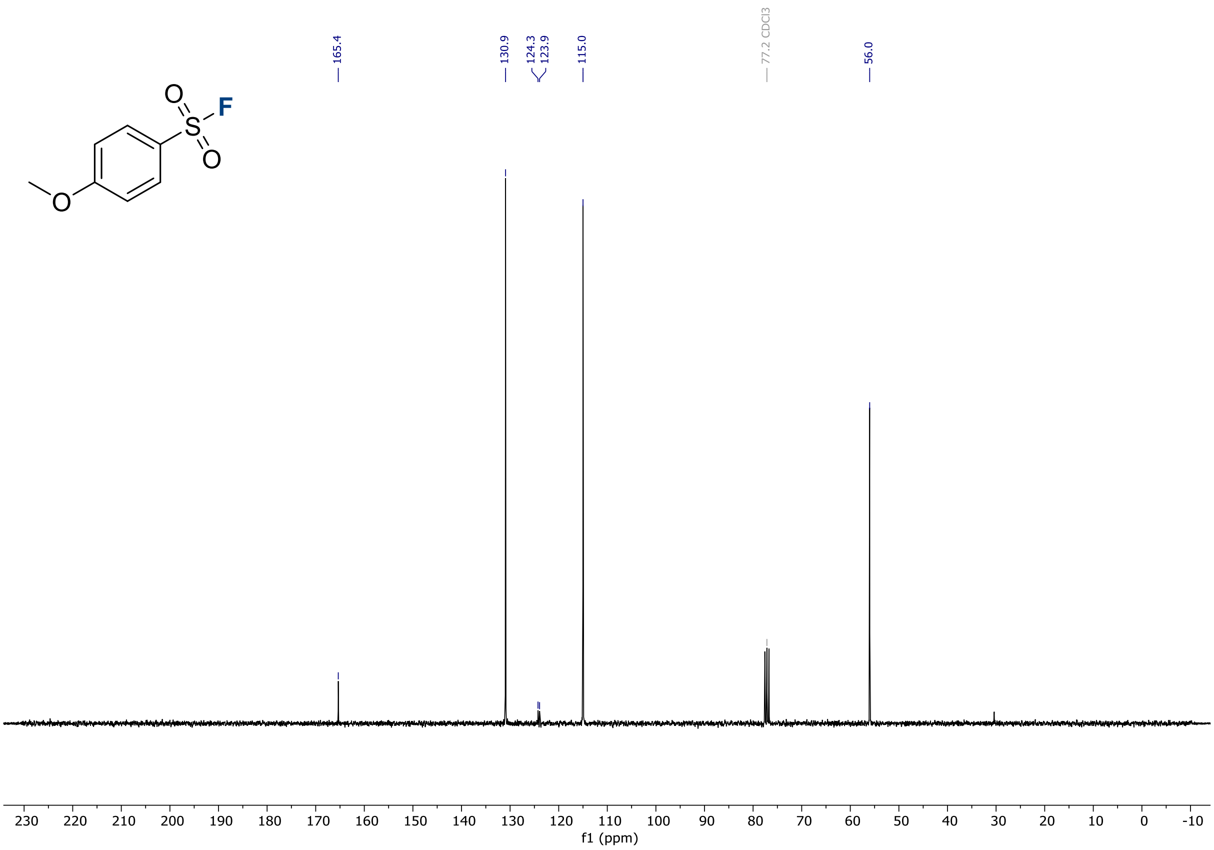

^{13}C NMR (75 MHz, $CDCl_3$) spectrum of **1a**.

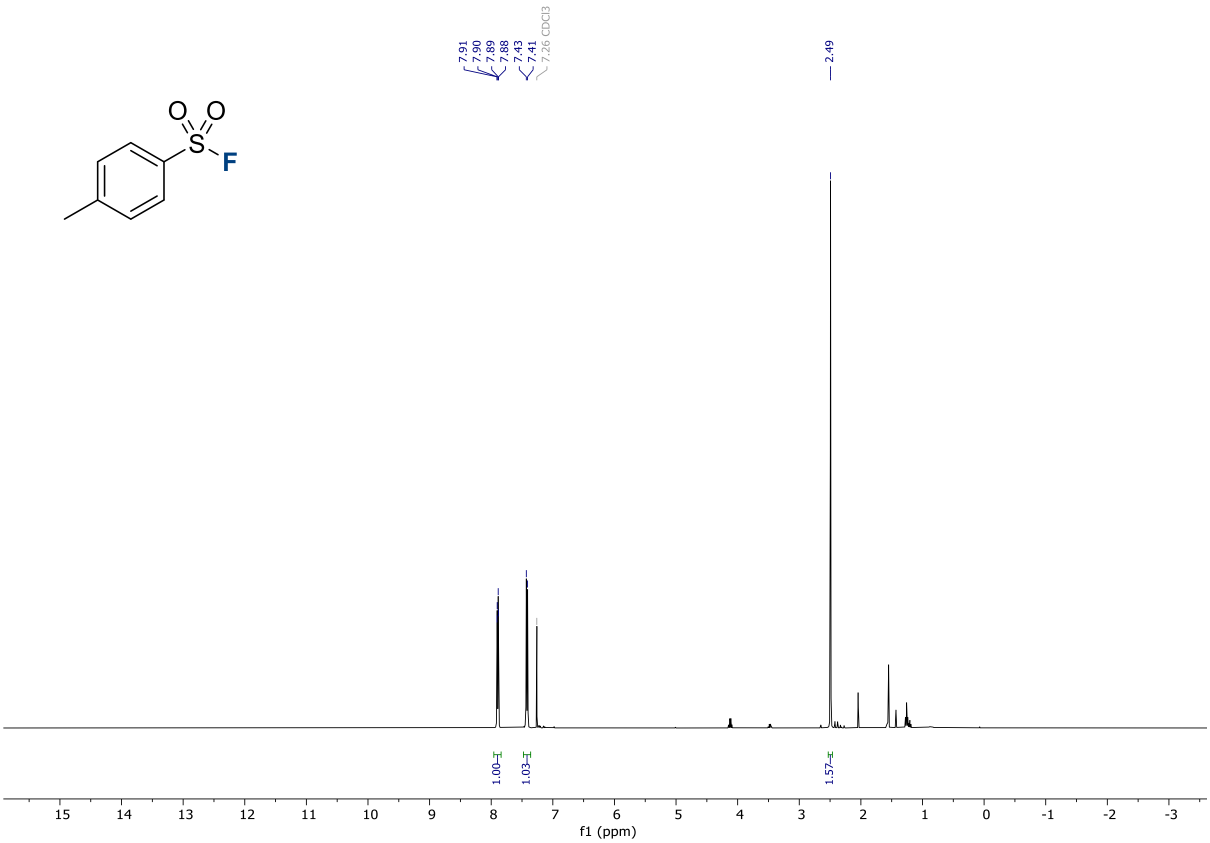

^{1}H NMR (400 MHz, $CDCl_3$, 298 K) spectrum of **1b**.

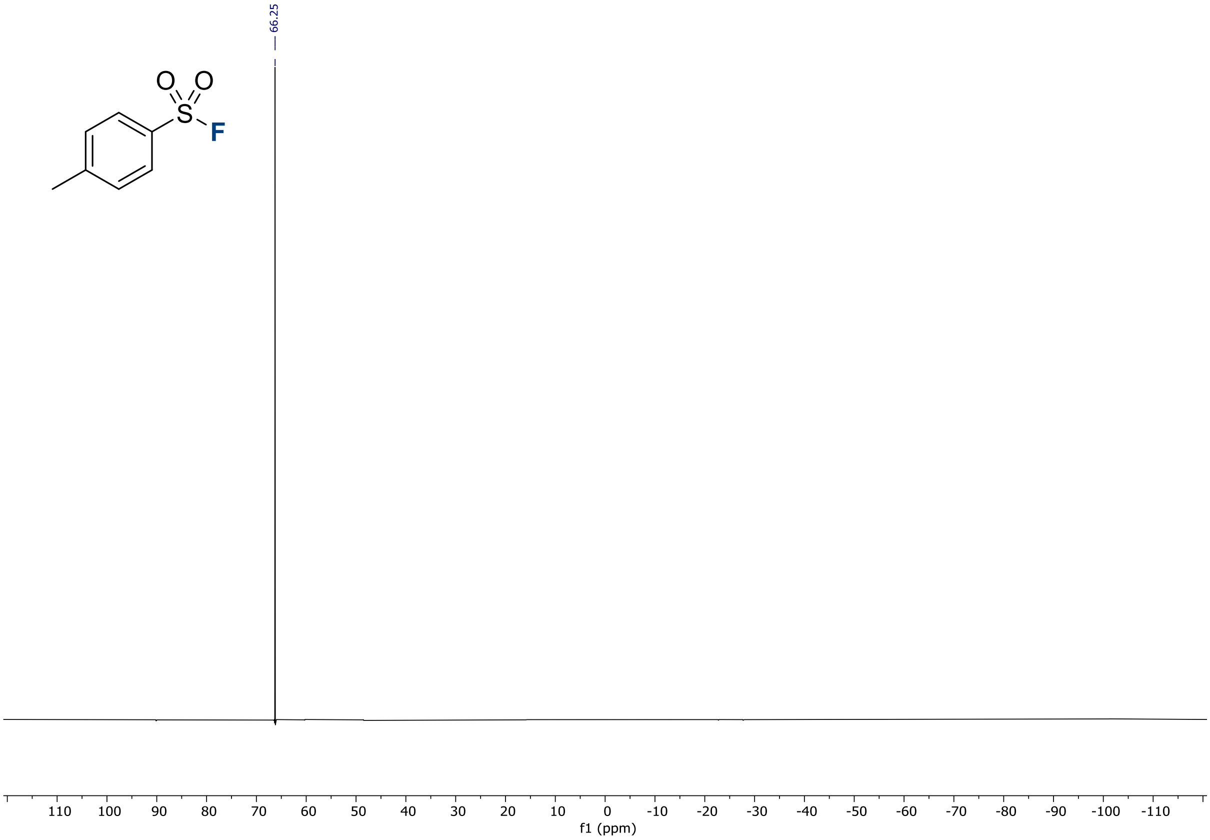

^{19}F NMR (376 MHz, $CDCl_3$, 298 K) spectrum of **1b**

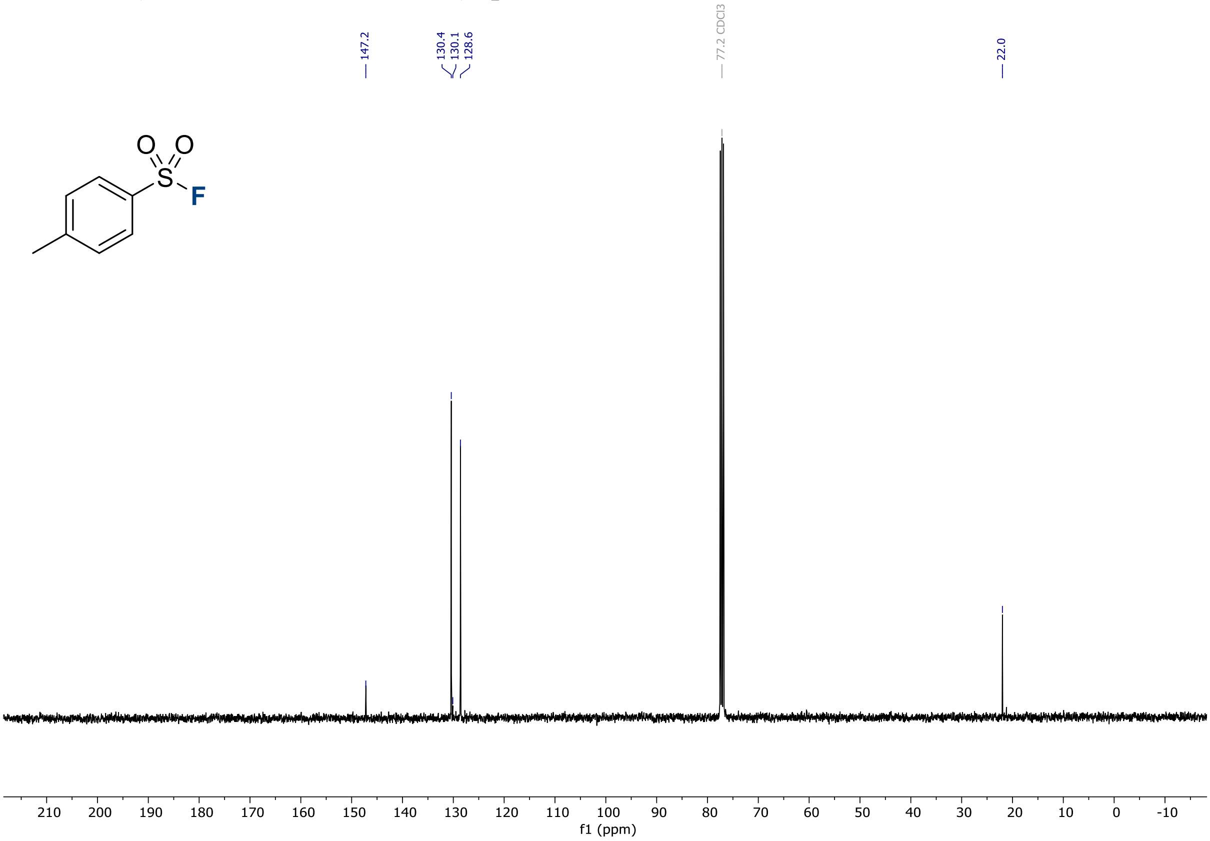

^{13}C NMR (101 MHz, $CDCl_3$, 298 K) spectrum of **1b**.

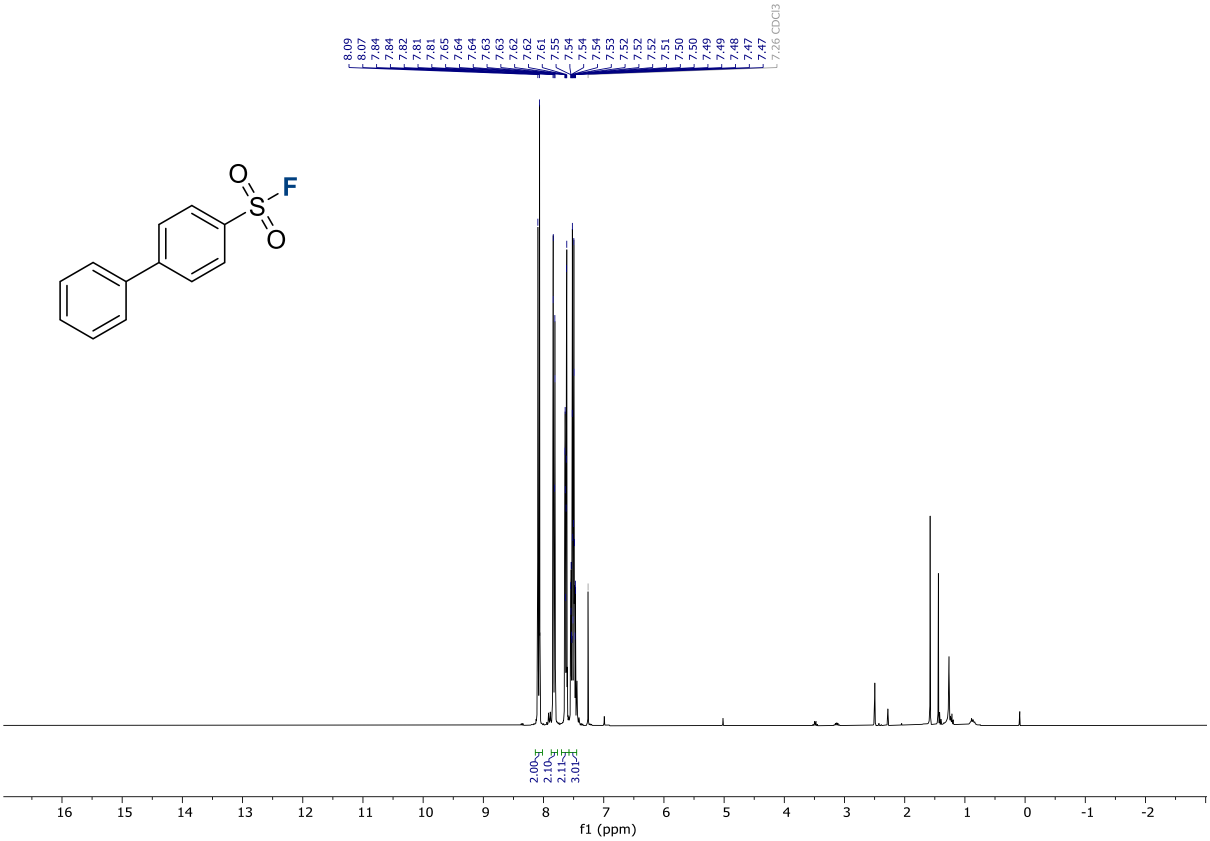

^{1}H NMR (400 MHz, $CDCl_3$, 298 K) spectrum of **1c**.

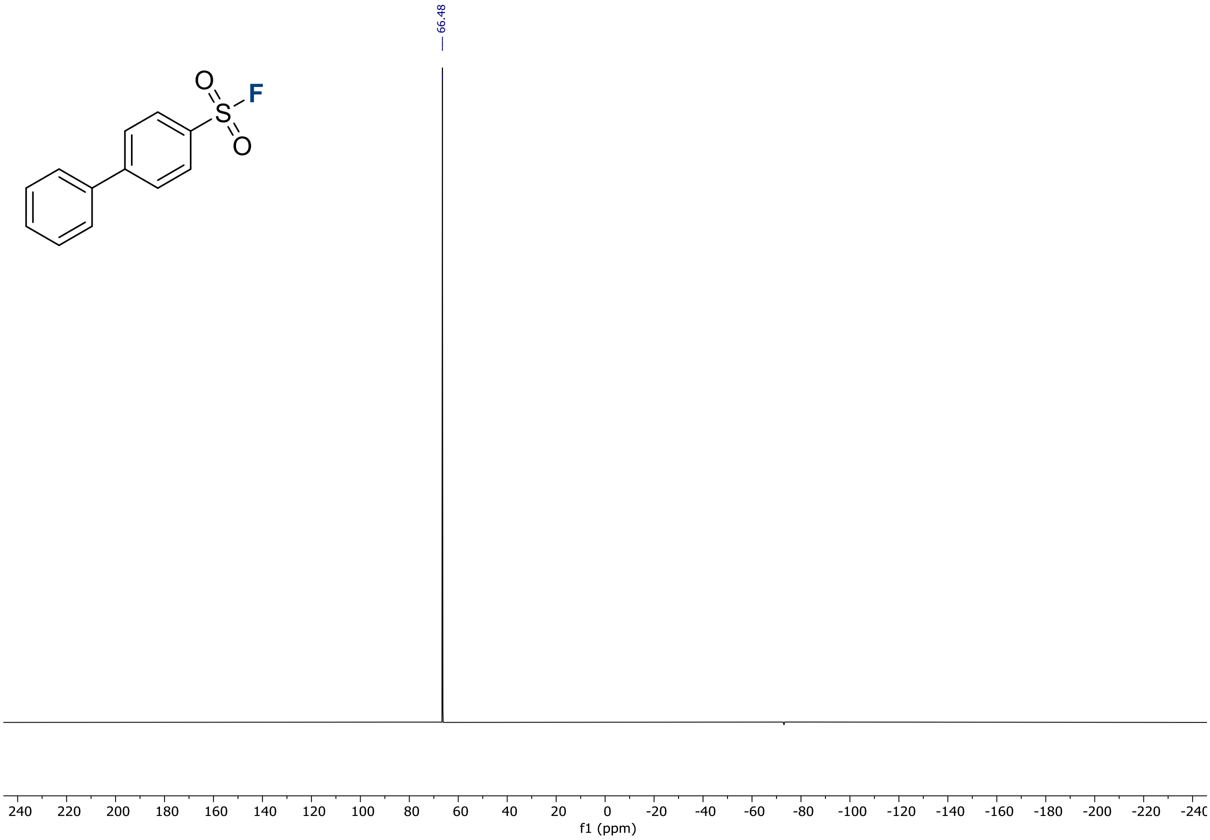

Proton-decoupled ^{19}F NMR (282 MHz, $CDCl_3$, 298 K) spectrum of **1c**.

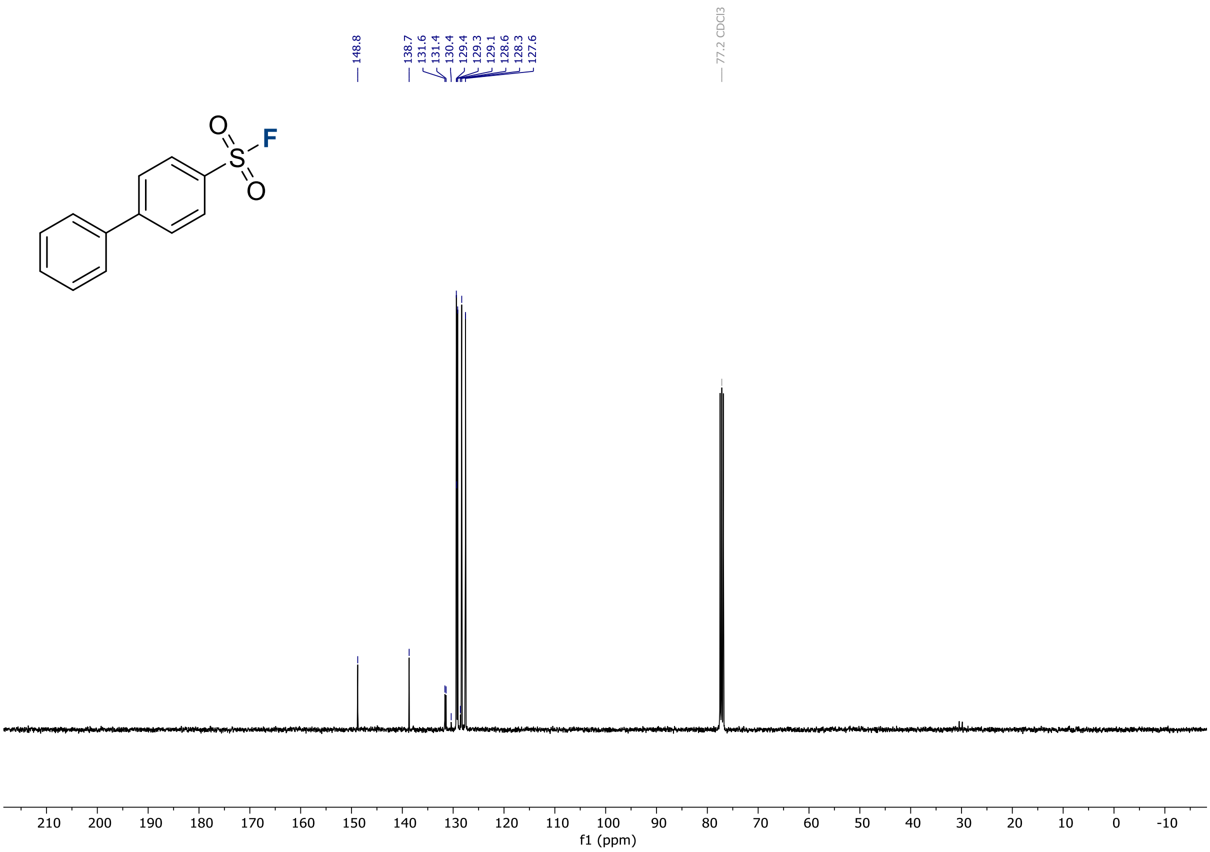

^{13}C NMR (101 MHz, CDCl$_3$, 298 K) spectrum of **1c**.

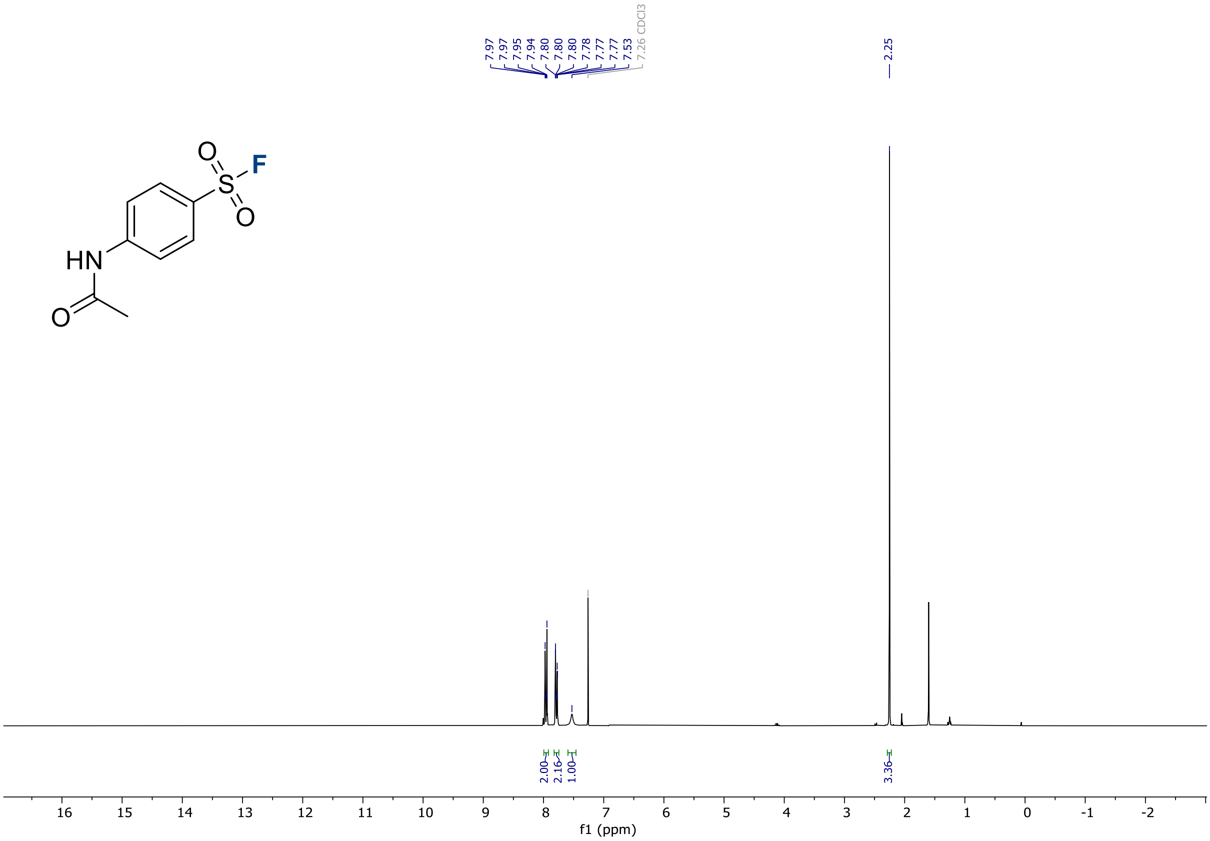

^{1}H NMR (300 MHz, CDCl$_3$, 298 K) spectrum of **1d**.

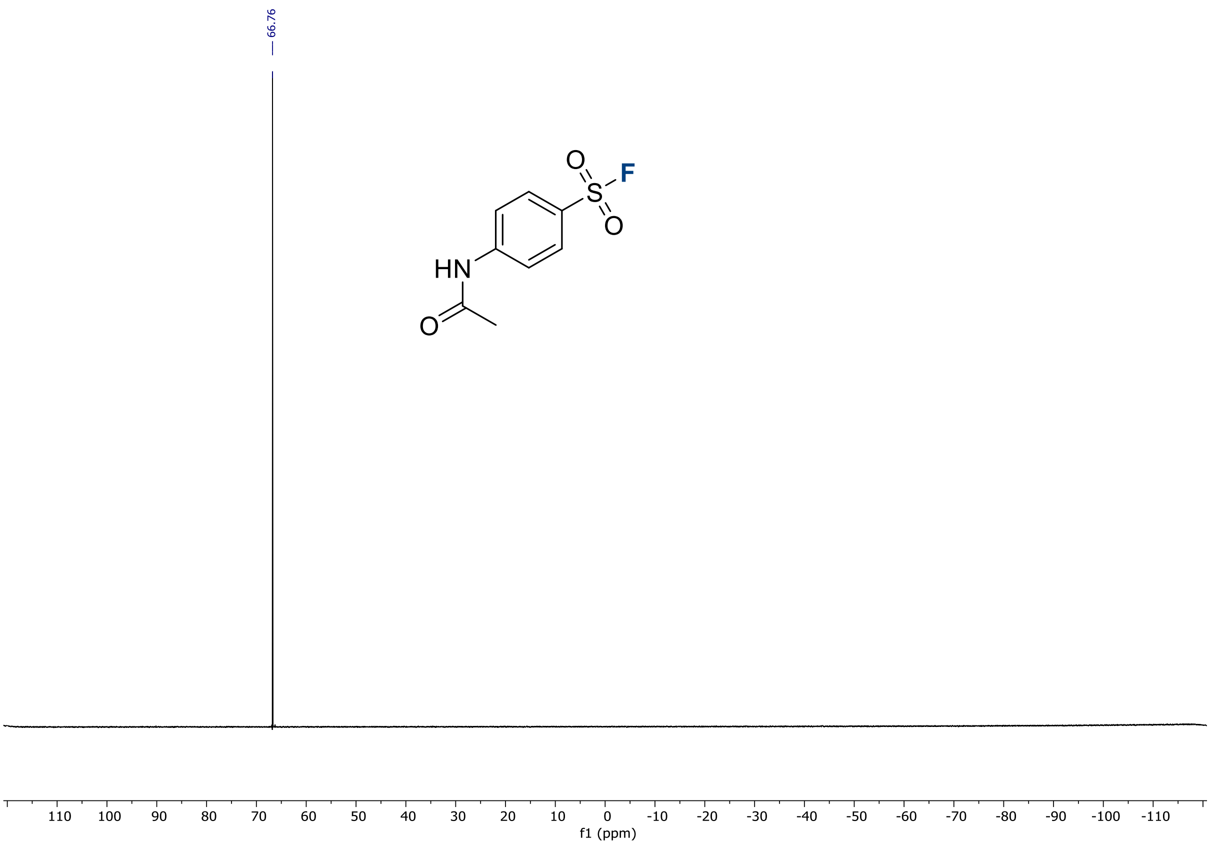

^{19}F NMR (376 MHz, $CDCl_3$, 298 K) spectrum of **1d**.

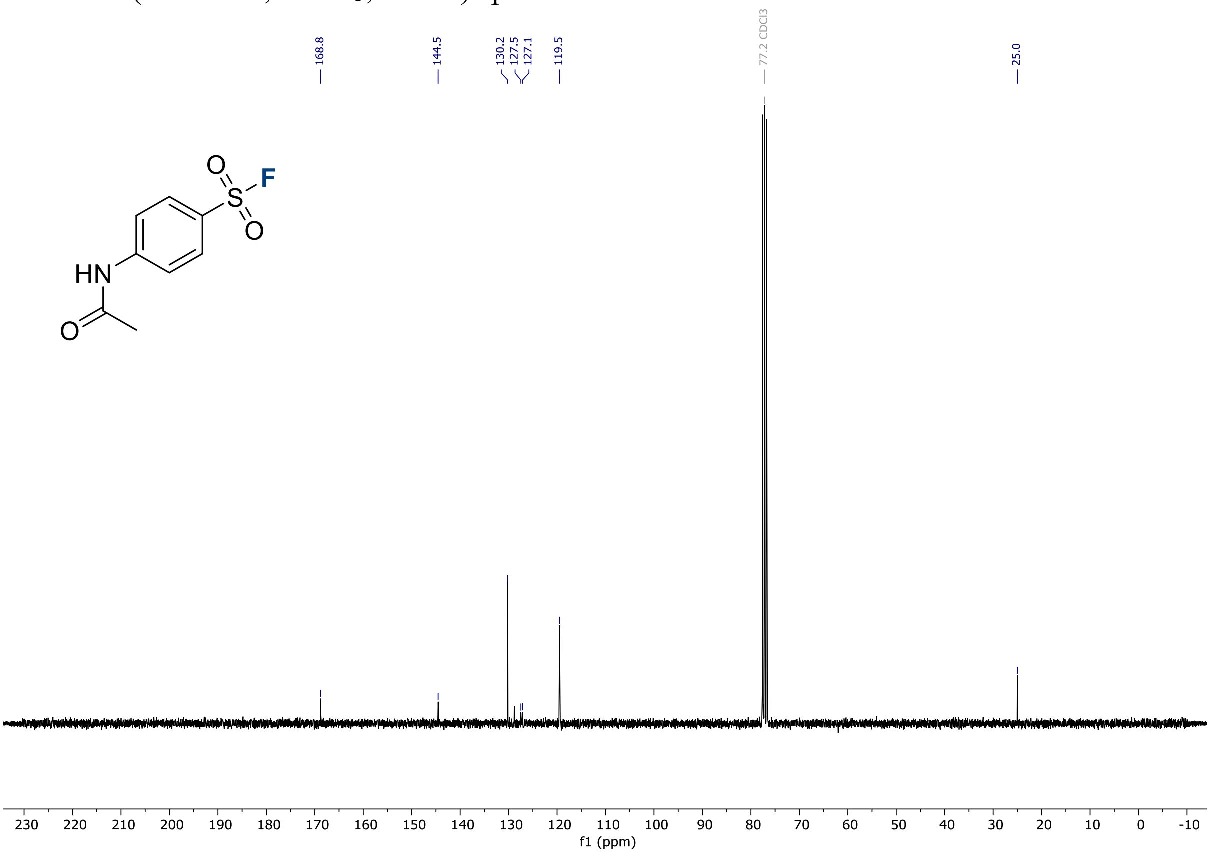

^{13}C NMR (75 MHz, $CDCl_3$, 298 K) spectrum of **1d**.

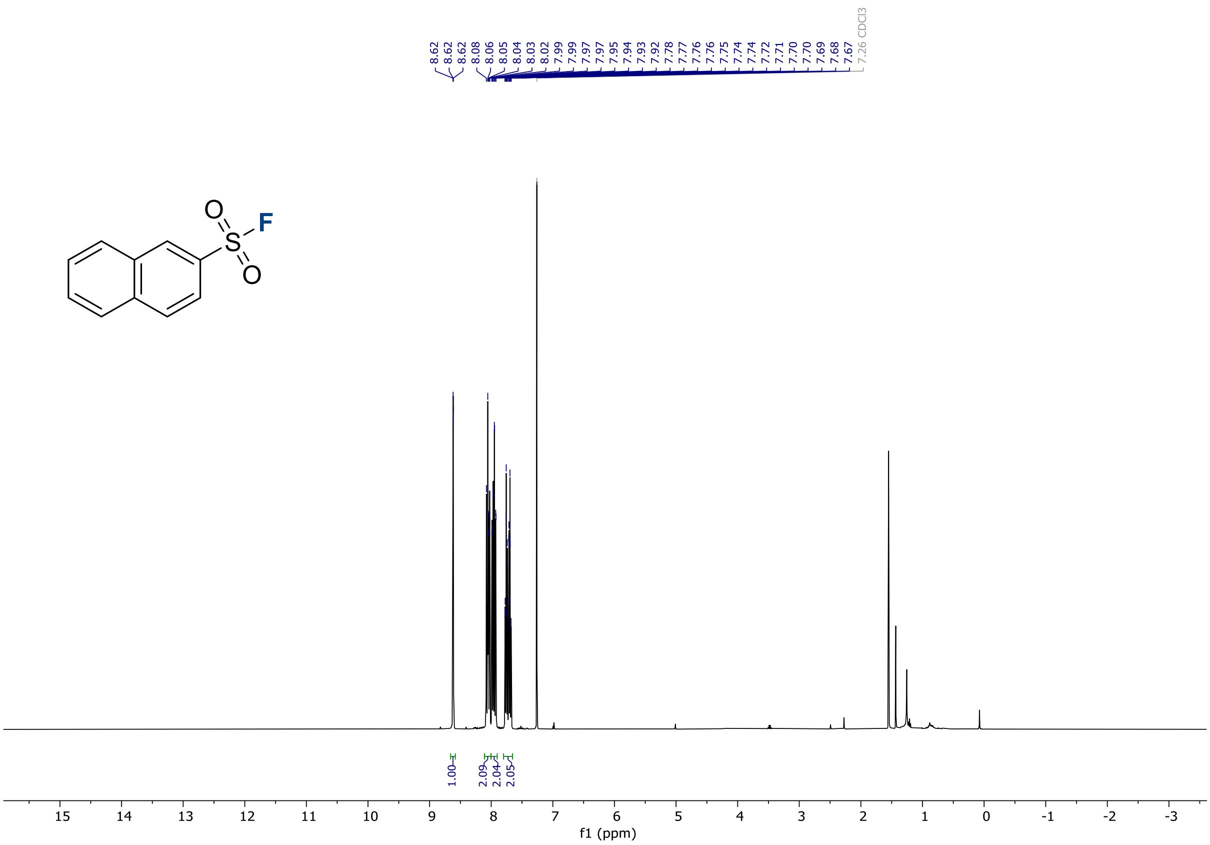

^1H NMR (400 MHz, $CDCl_3$, 298 K) spectrum of **1e**.

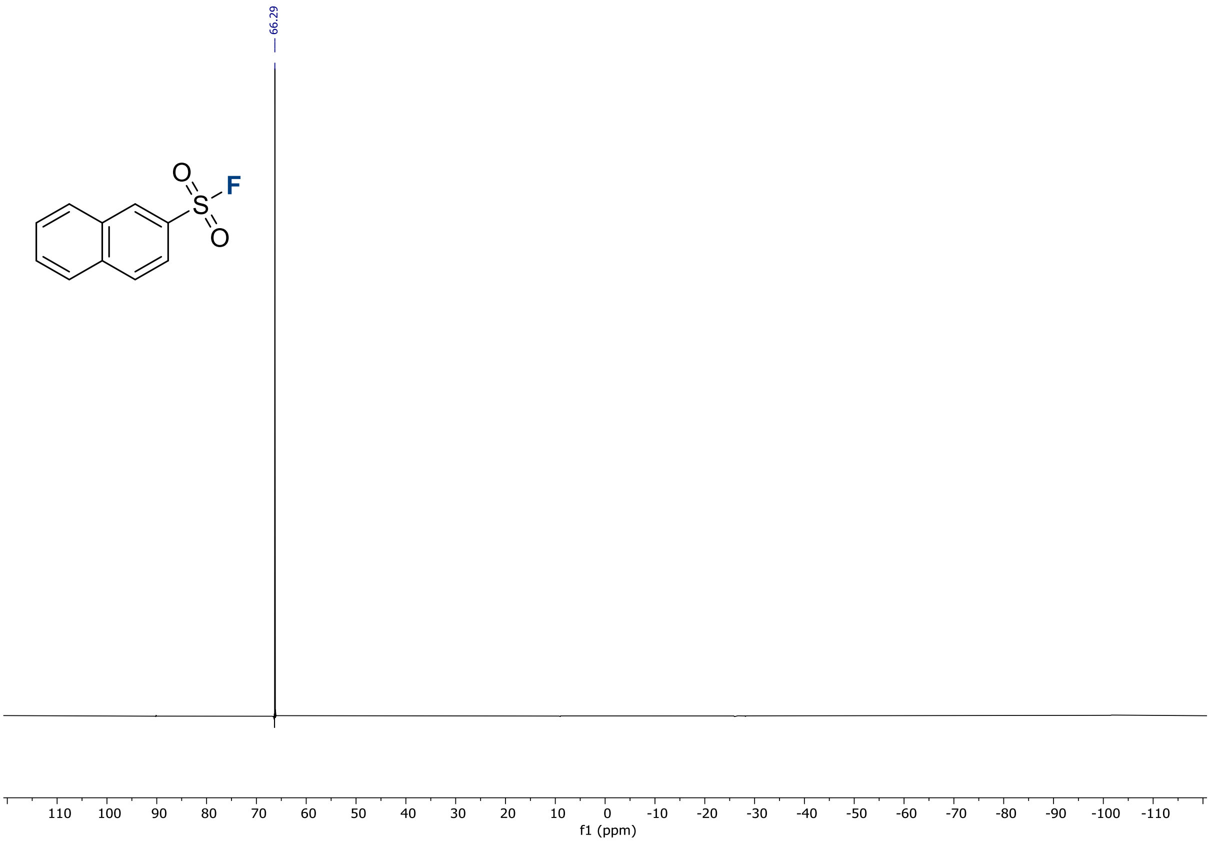

^{19}F NMR (376 MHz, $CDCl_3$, 298 K) spectrum of **1e**.

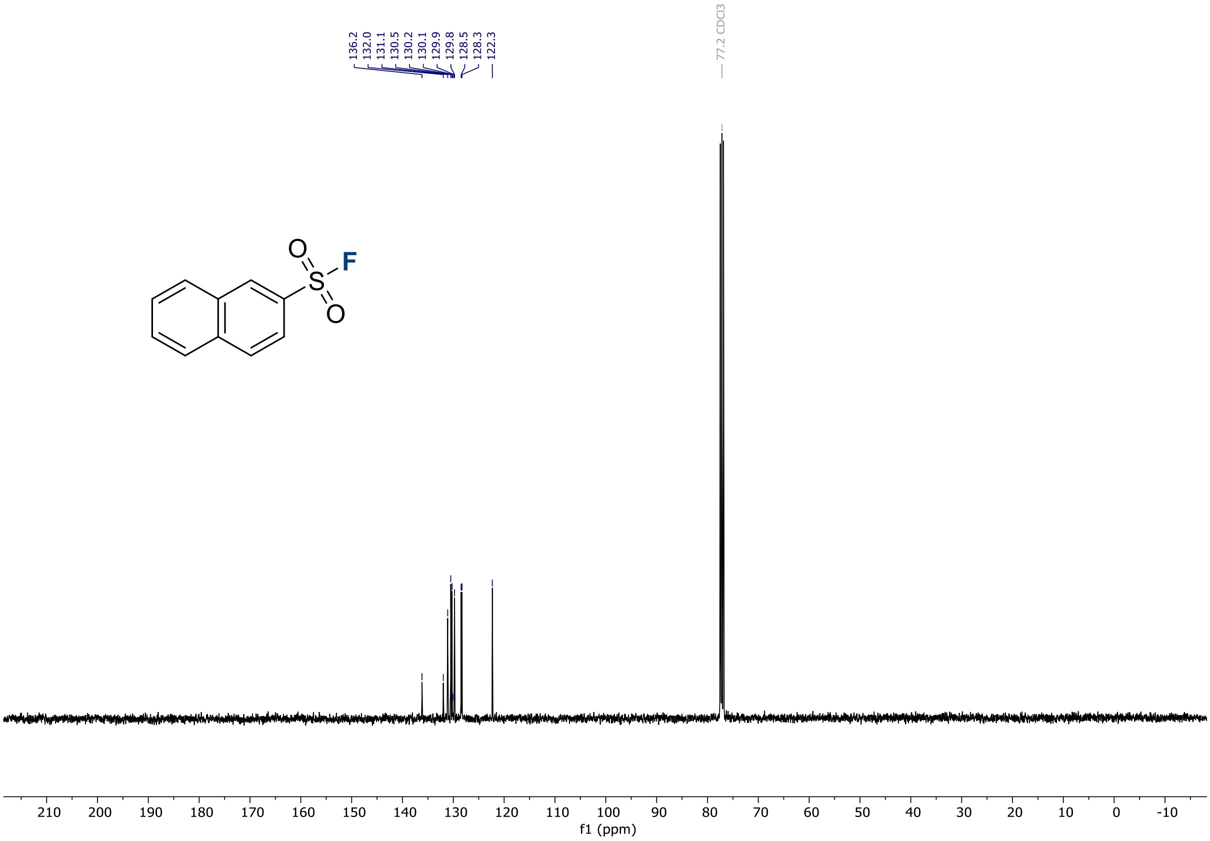

^{13}C NMR (75 MHz, $CDCl_3$, 298 K) spectrum of **1e**.

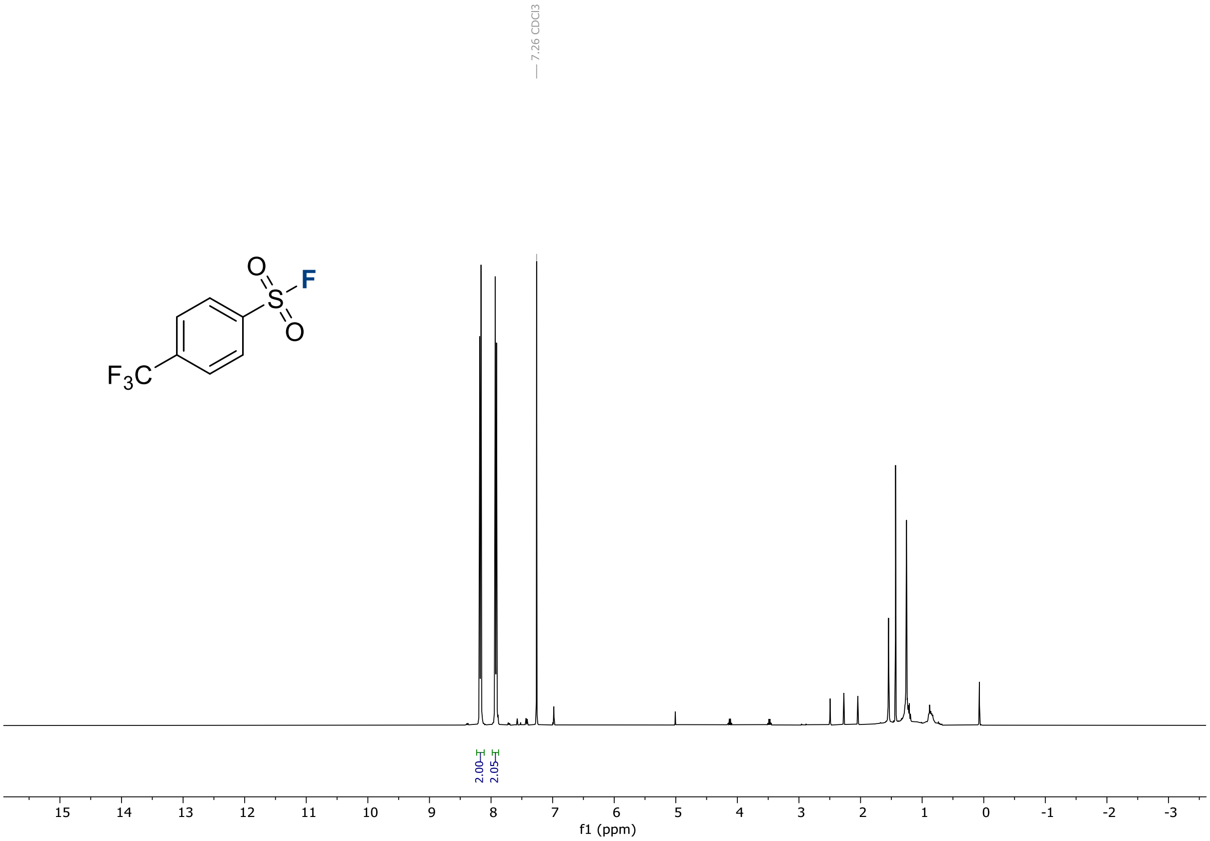

^{1}H NMR (400 MHz, $CDCl_3$, 298 K) spectrum of **1f**.

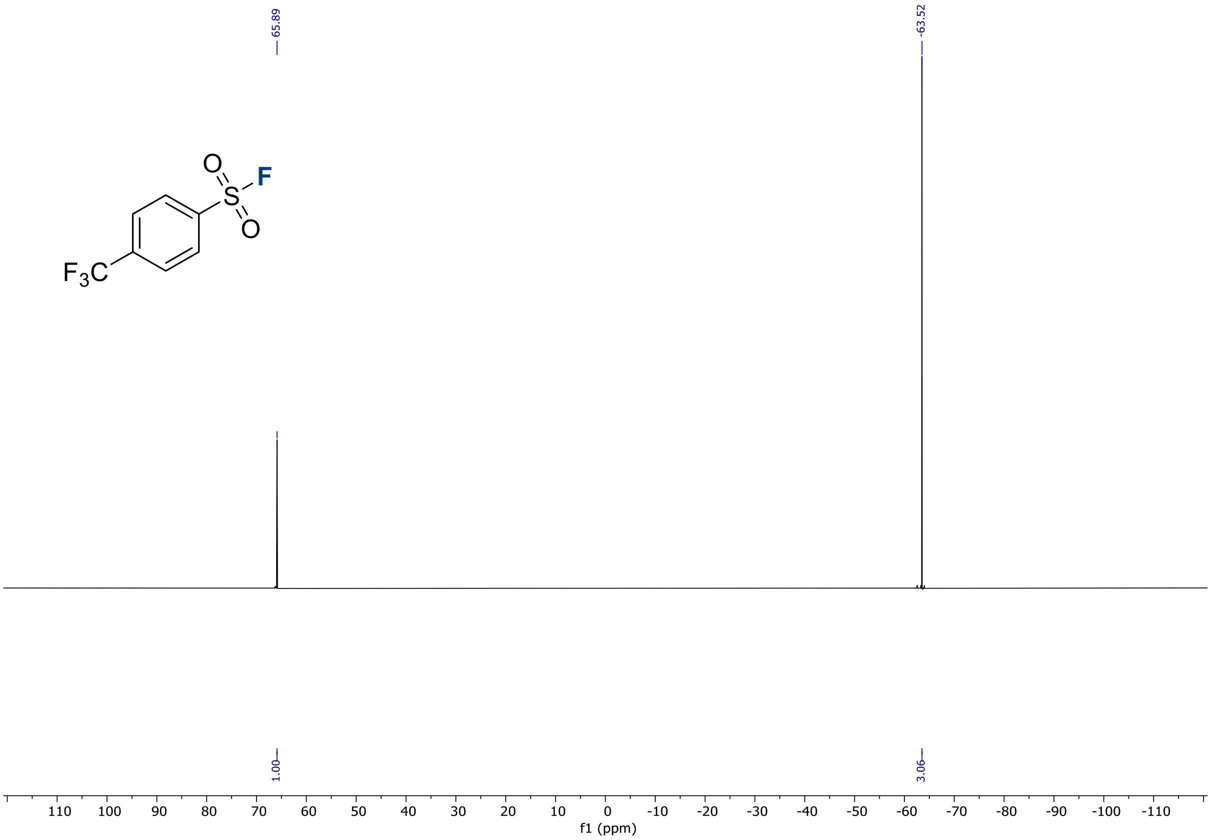

^{19}F NMR (376 MHz, $CDCl_3$, 298 K) spectrum of **1f**.

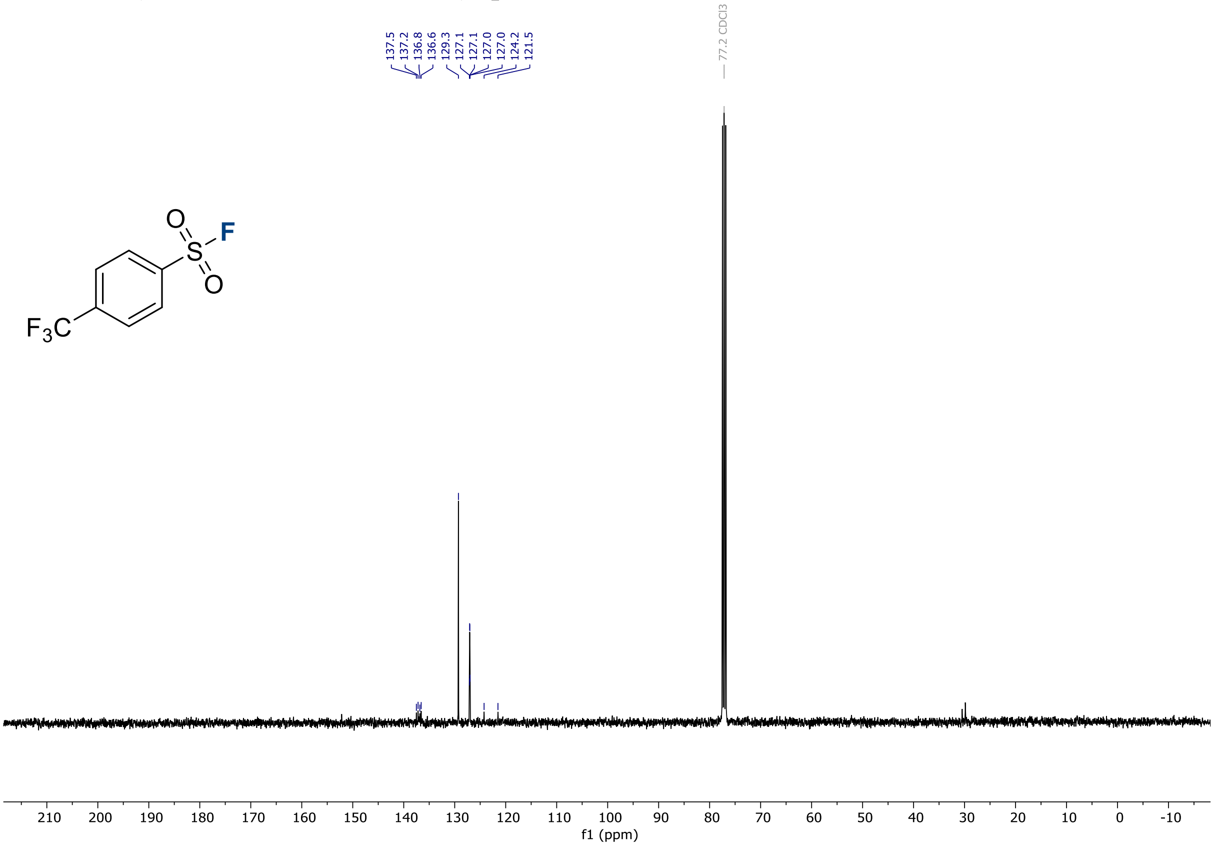

^{13}C NMR (101 MHz, $CDCl_3$, 298 K) spectrum of **1f**.

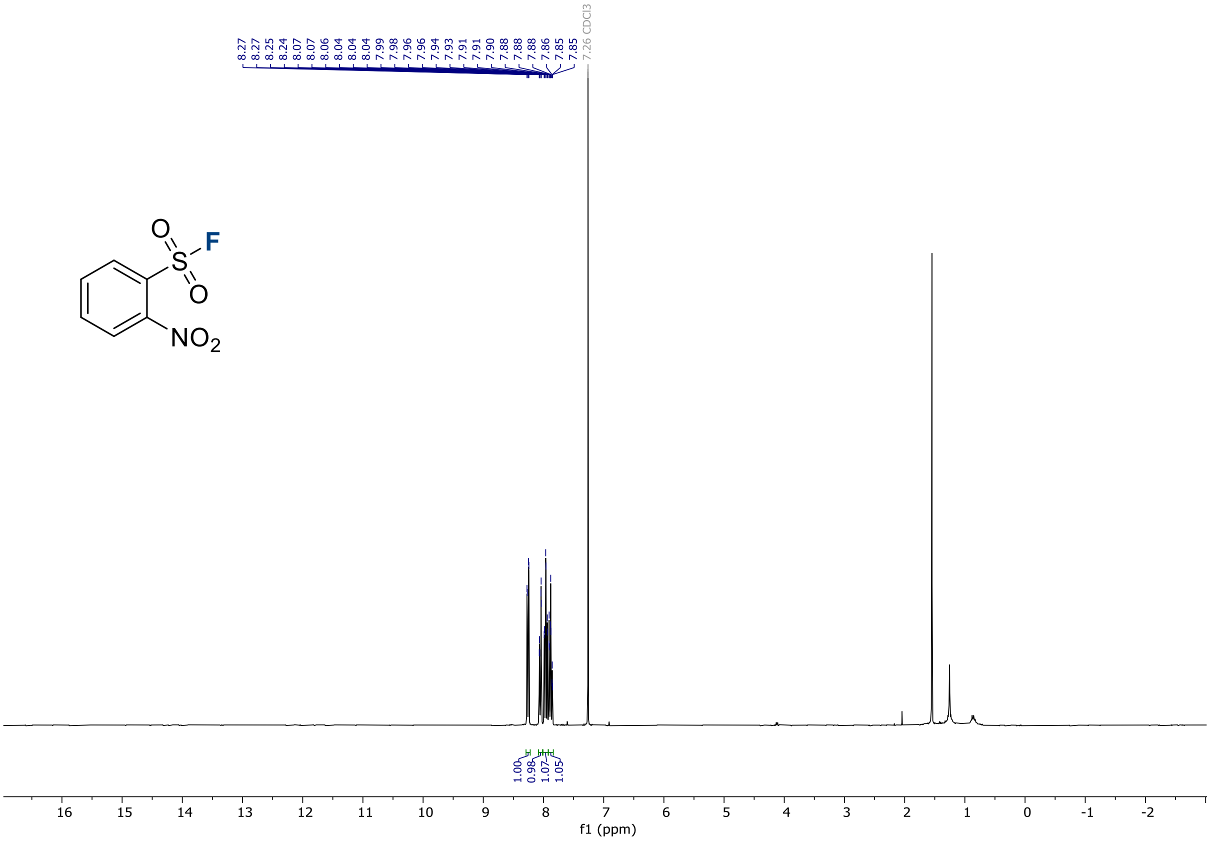

^{1}H NMR (300 MHz, $CDCl_3$, 298 K) spectrum of **1g**.

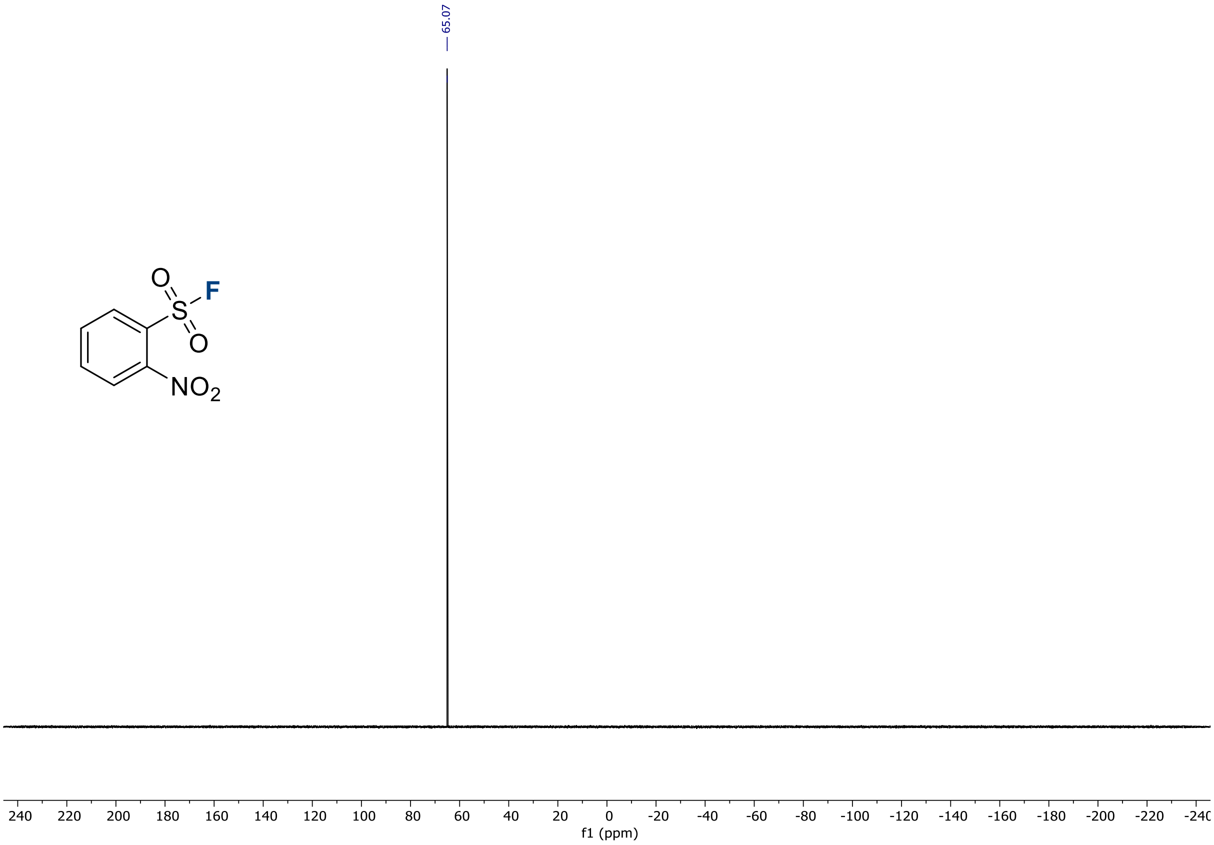

^{19}F NMR (282 MHz, $CDCl_3$, 298 K) spectrum of **1g**.

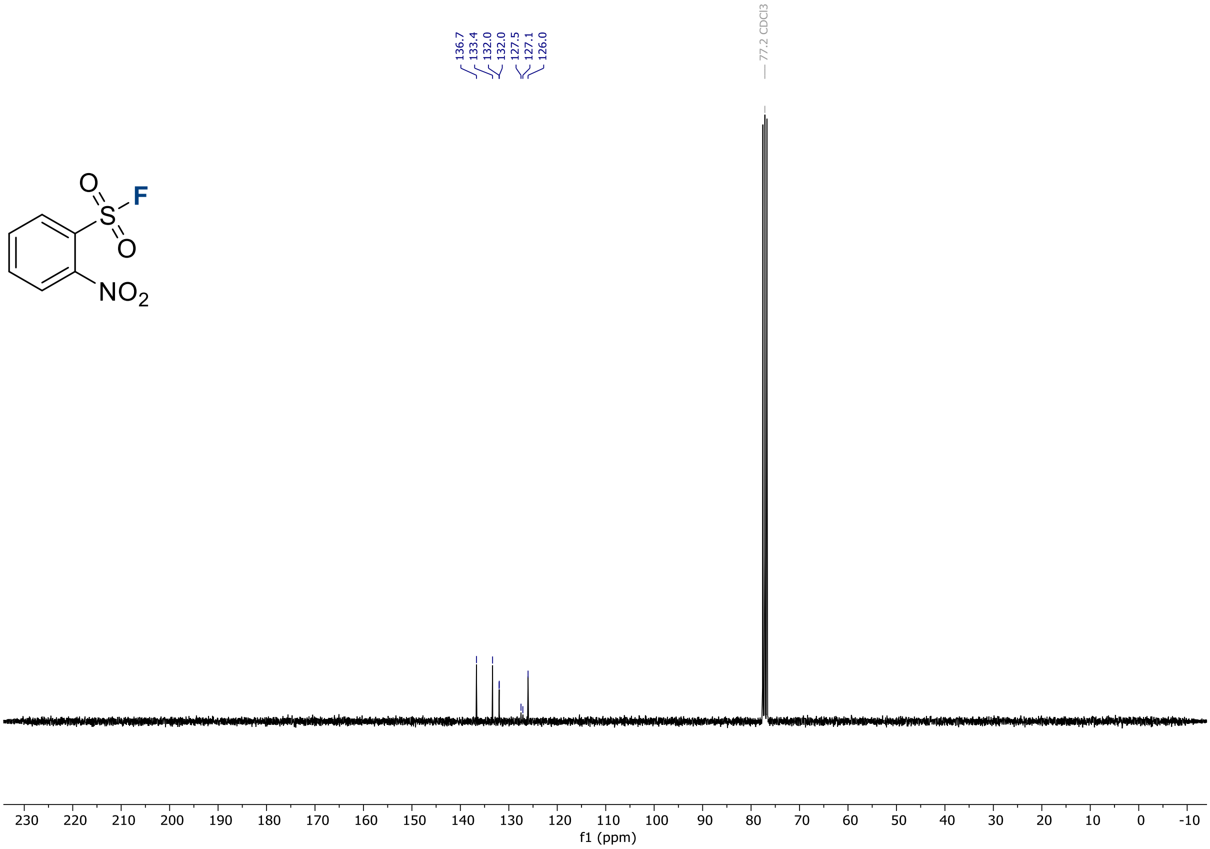

^{13}C NMR (75 MHz, $CDCl_3$, 298 K) spectrum of **1g**.

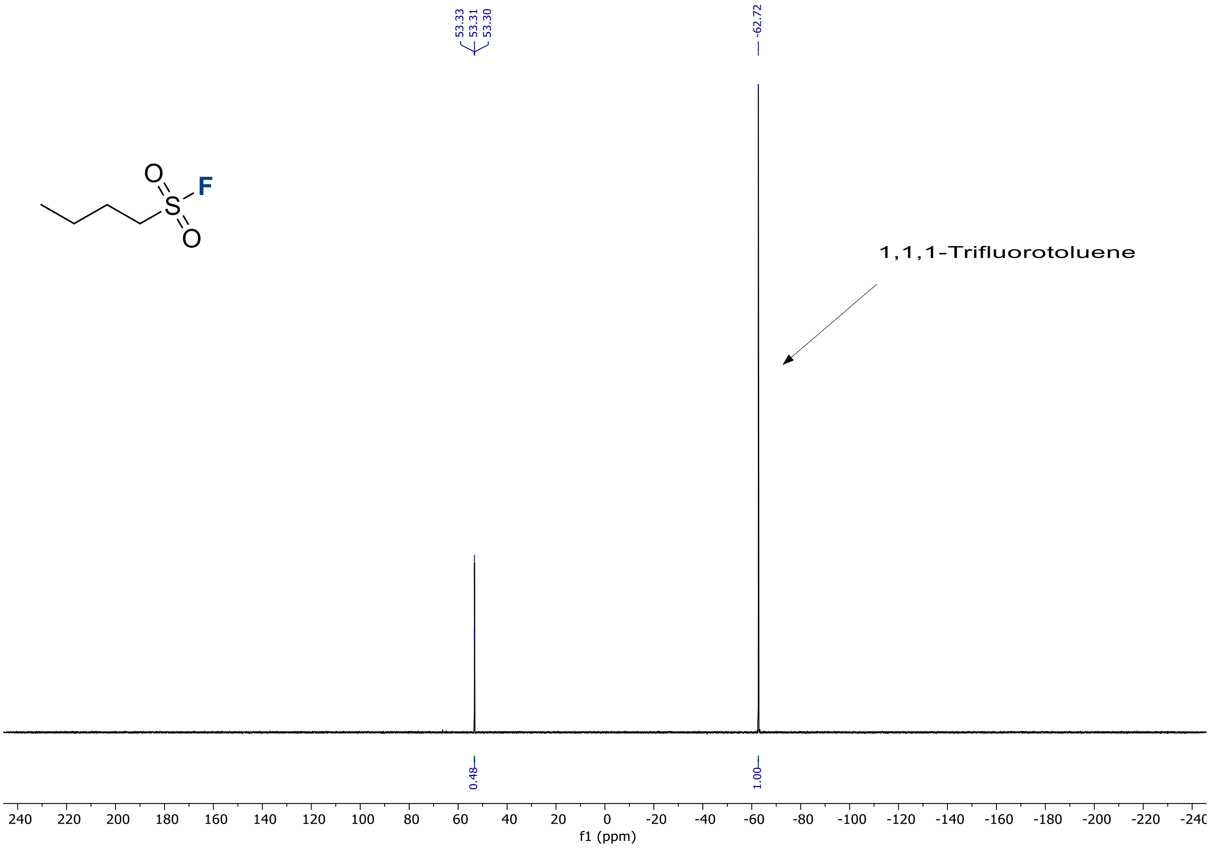

Crude, quantitative, ^{19}F NMR (282 MHz, $CDCl_3$, 298 K) spectrum of **1h** with 1,1,1-trifluorotoluene (0.33 eq.) added as internal standard.

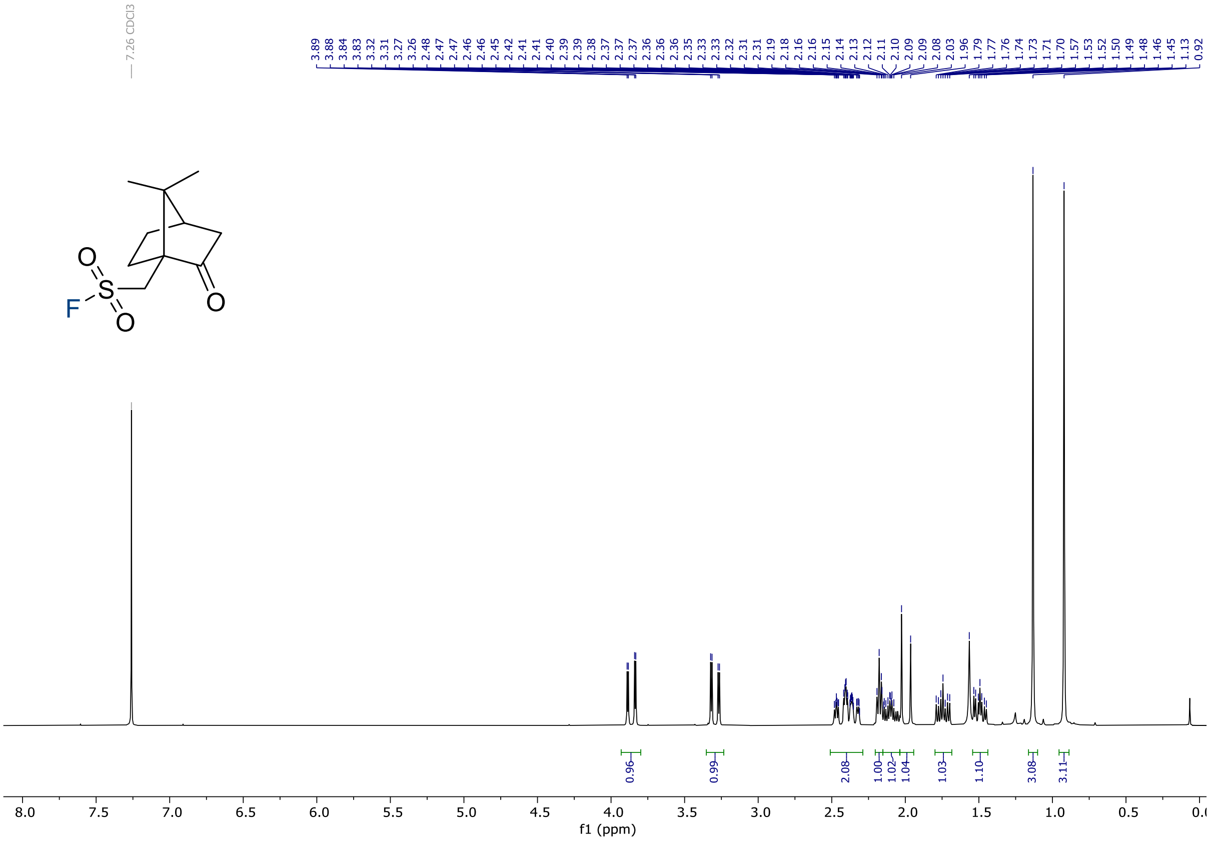

^{1}H NMR (400 MHz, $CDCl_3$, 298 K) spectrum of **1i**.

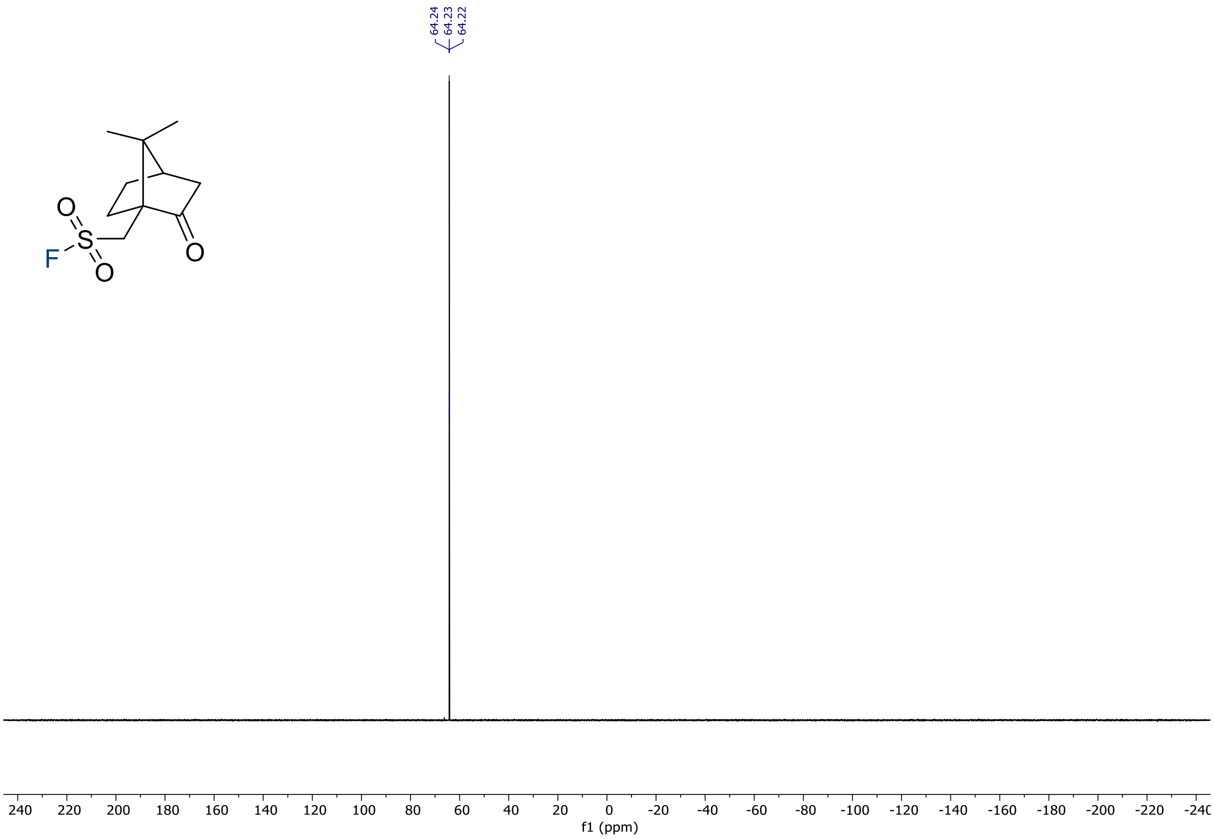

^{19}F NMR (282 MHz, $CDCl_3$, 298 K) spectrum of **1i**.

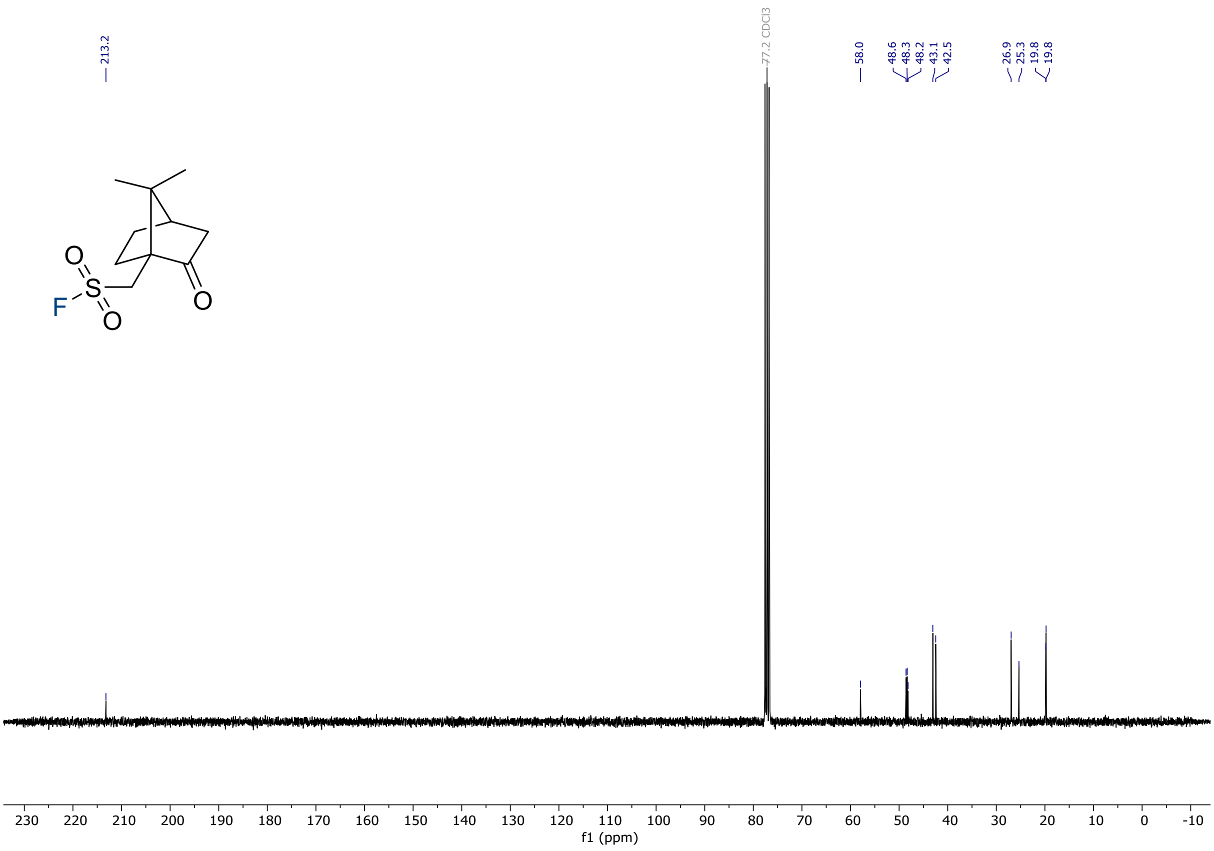

^{13}C NMR (101 MHz, $CDCl_3$, 298 K) spectrum of **1i**.

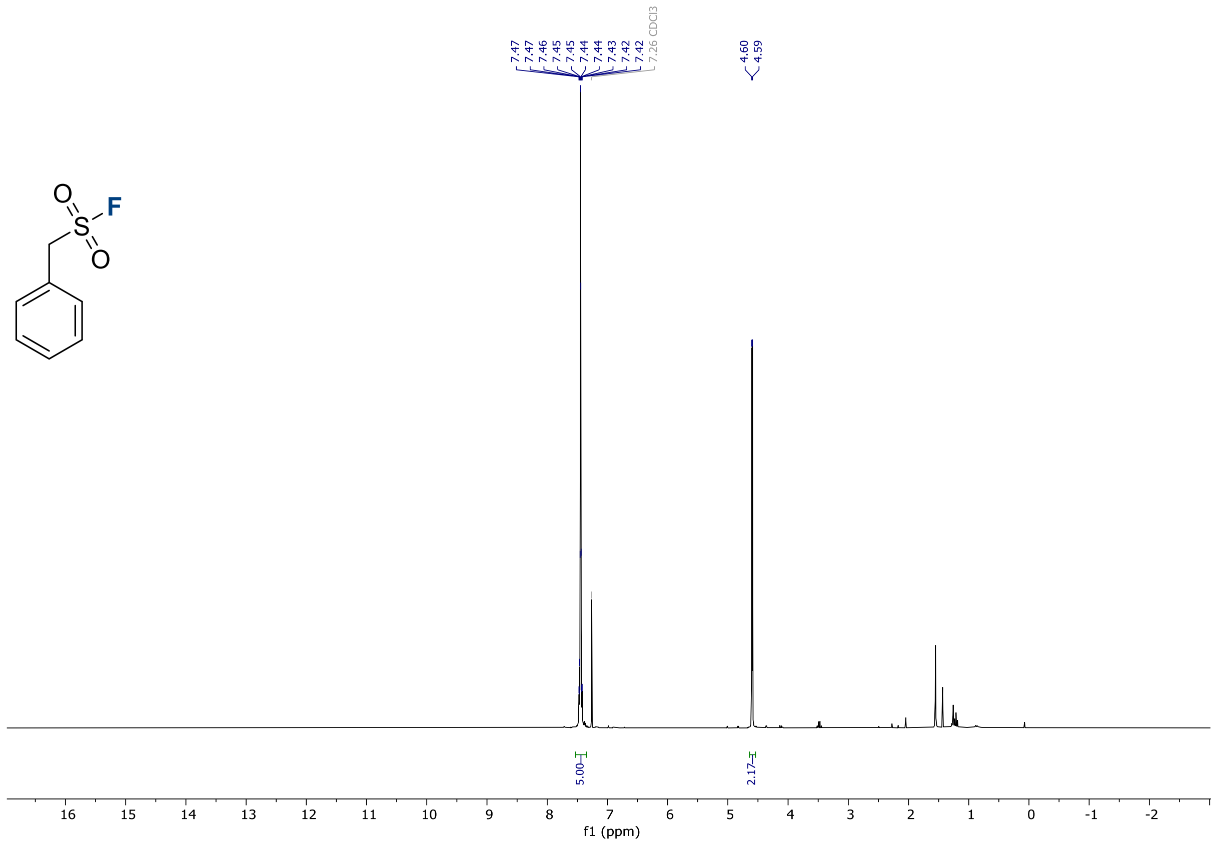

^{1}H NMR (300 MHz, $CDCl_3$, 298 K) spectrum of **1j**.

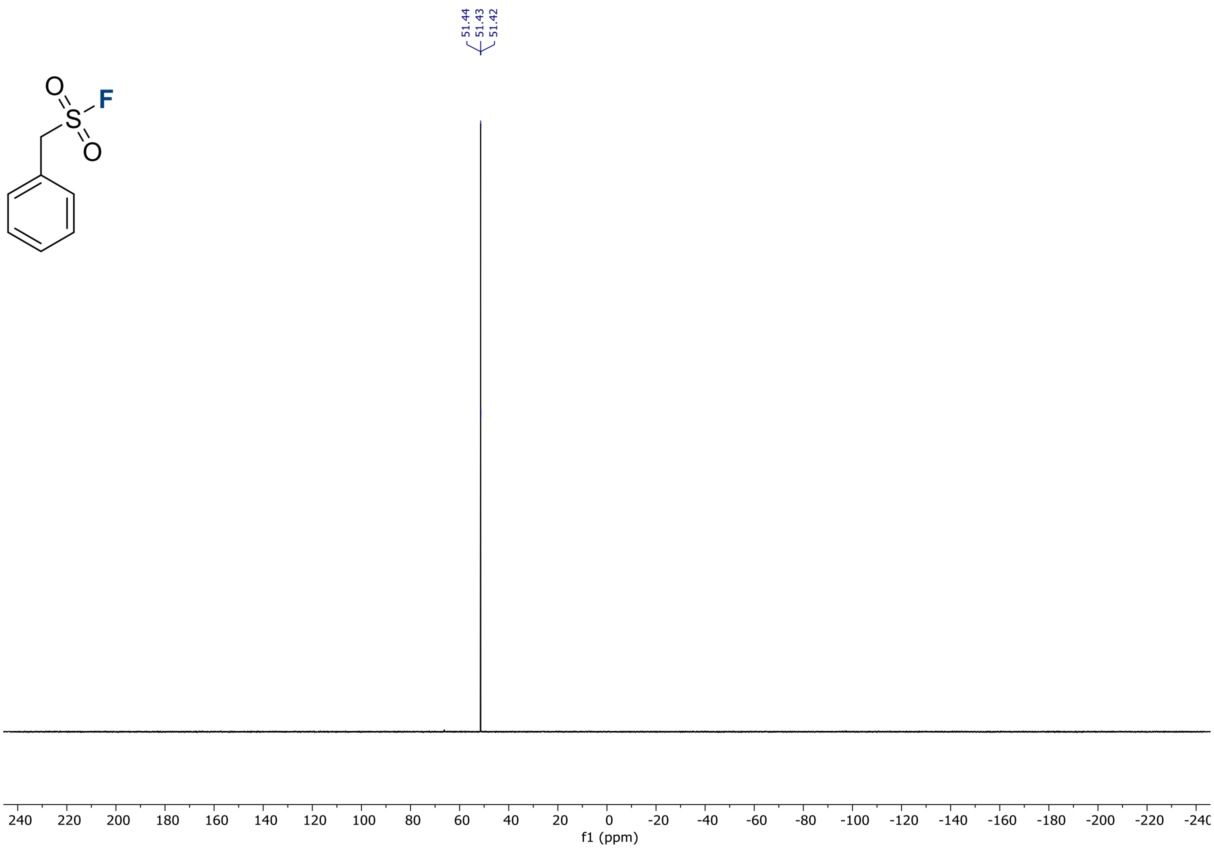

^{19}F NMR (282 MHz, $CDCl_3$, 298 K) spectrum of **1j**.

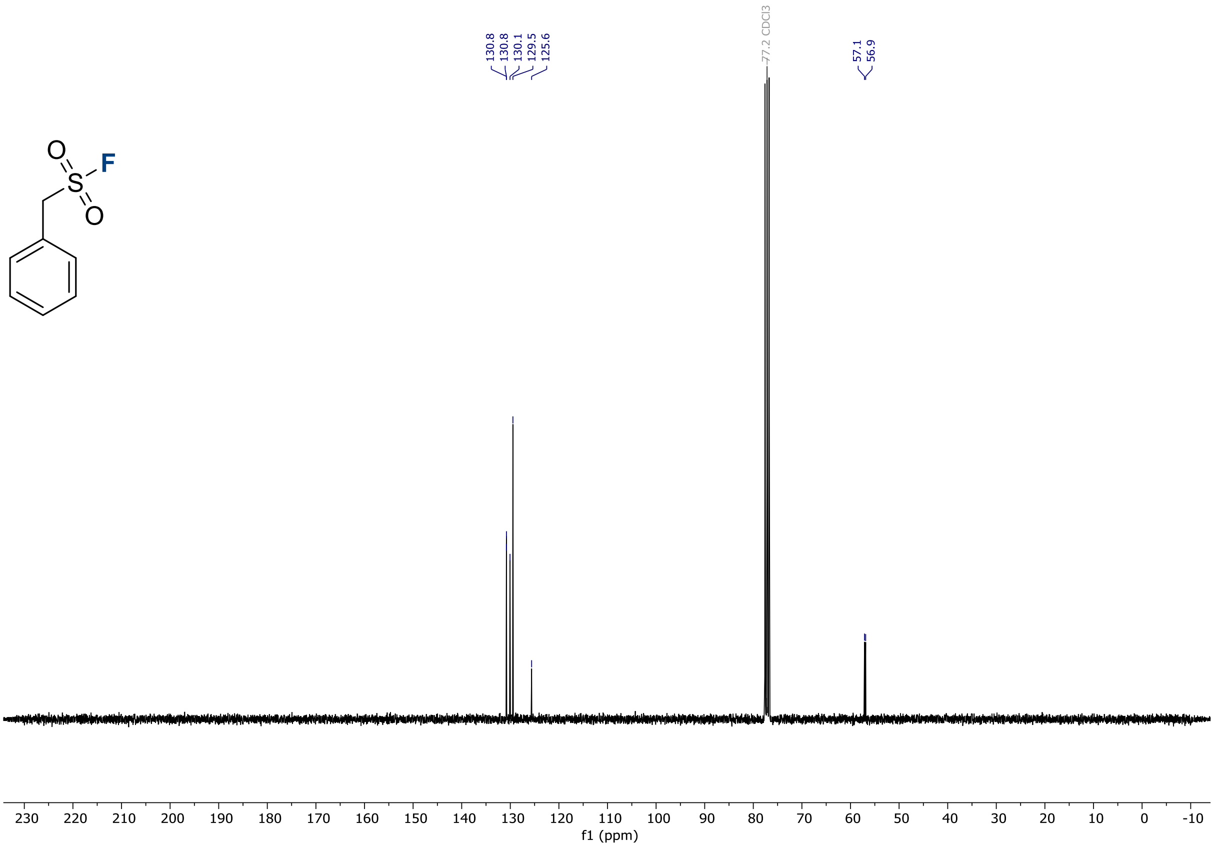

^{13}C NMR (101 MHz, $CDCl_3$, 298 K) spectrum of **1j**.

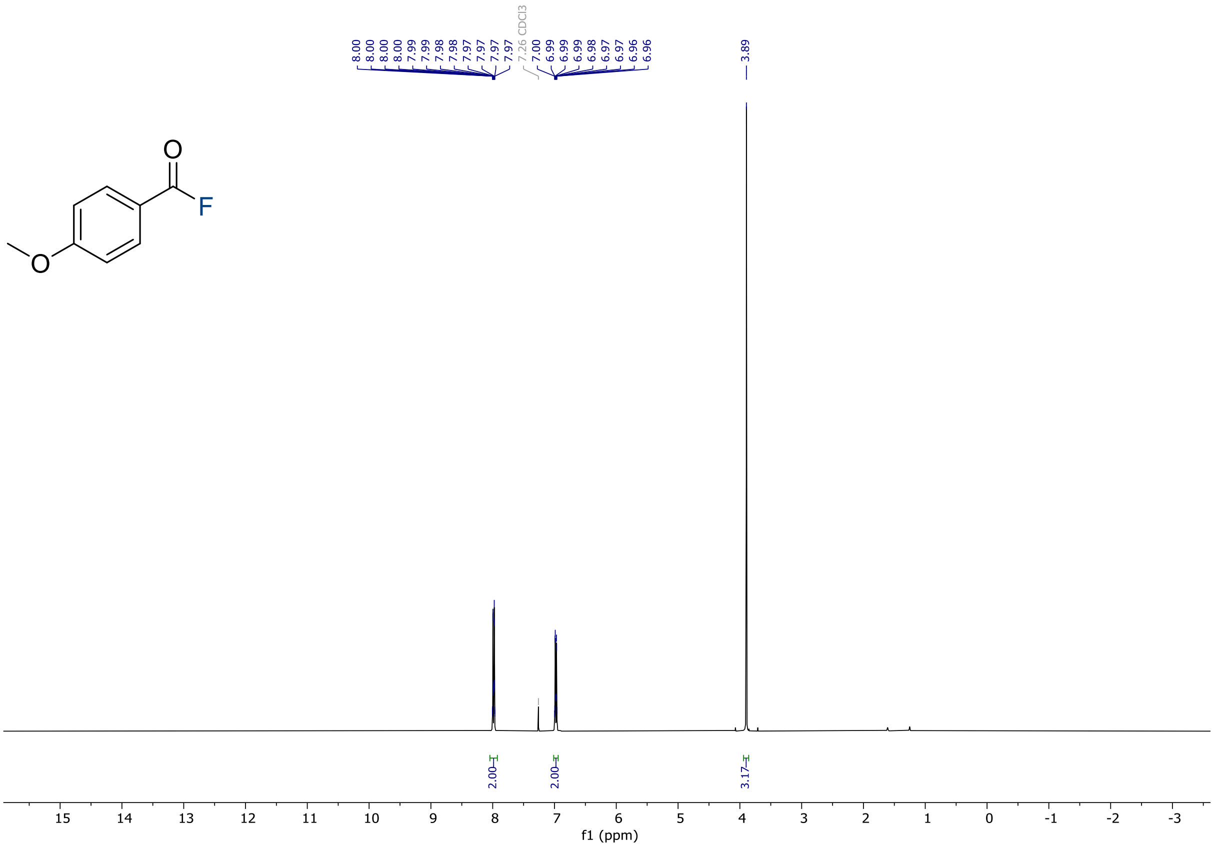

^{1}H NMR (400 MHz, $CDCl_3$, 298 K) spectrum of **1k**.

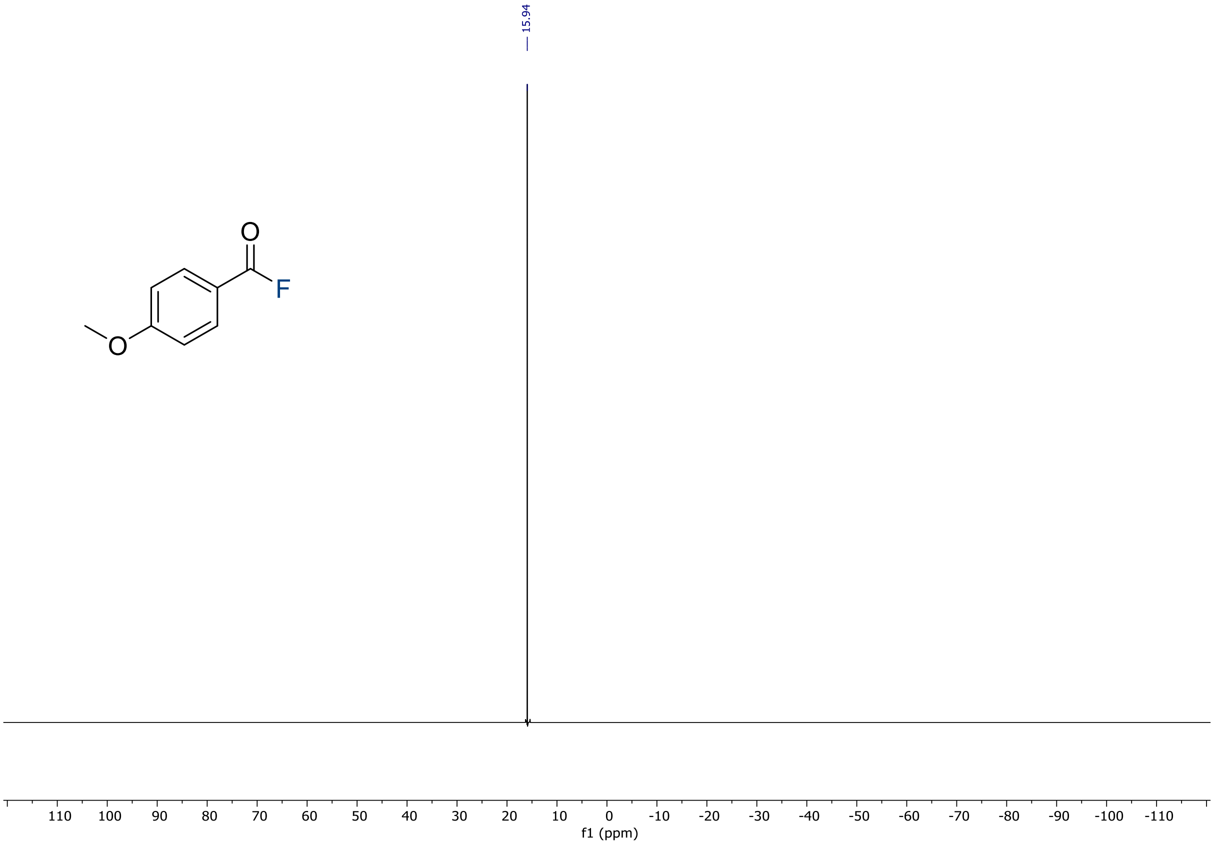

^{19}F NMR (376 MHz, $CDCl_3$, 298 K) spectrum of **1k**.

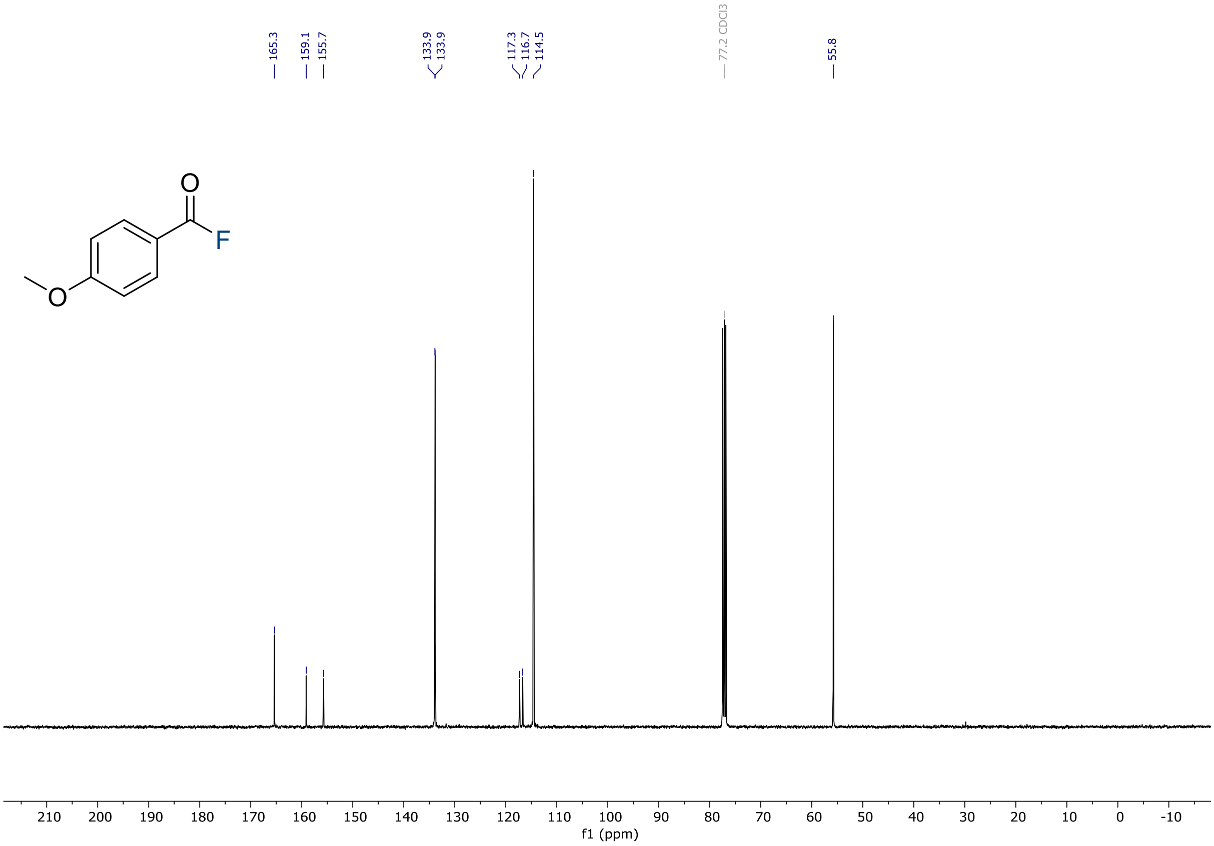

^{13}C NMR (101 MHz, $CDCl_3$, 298 K) spectrum of **1k**.

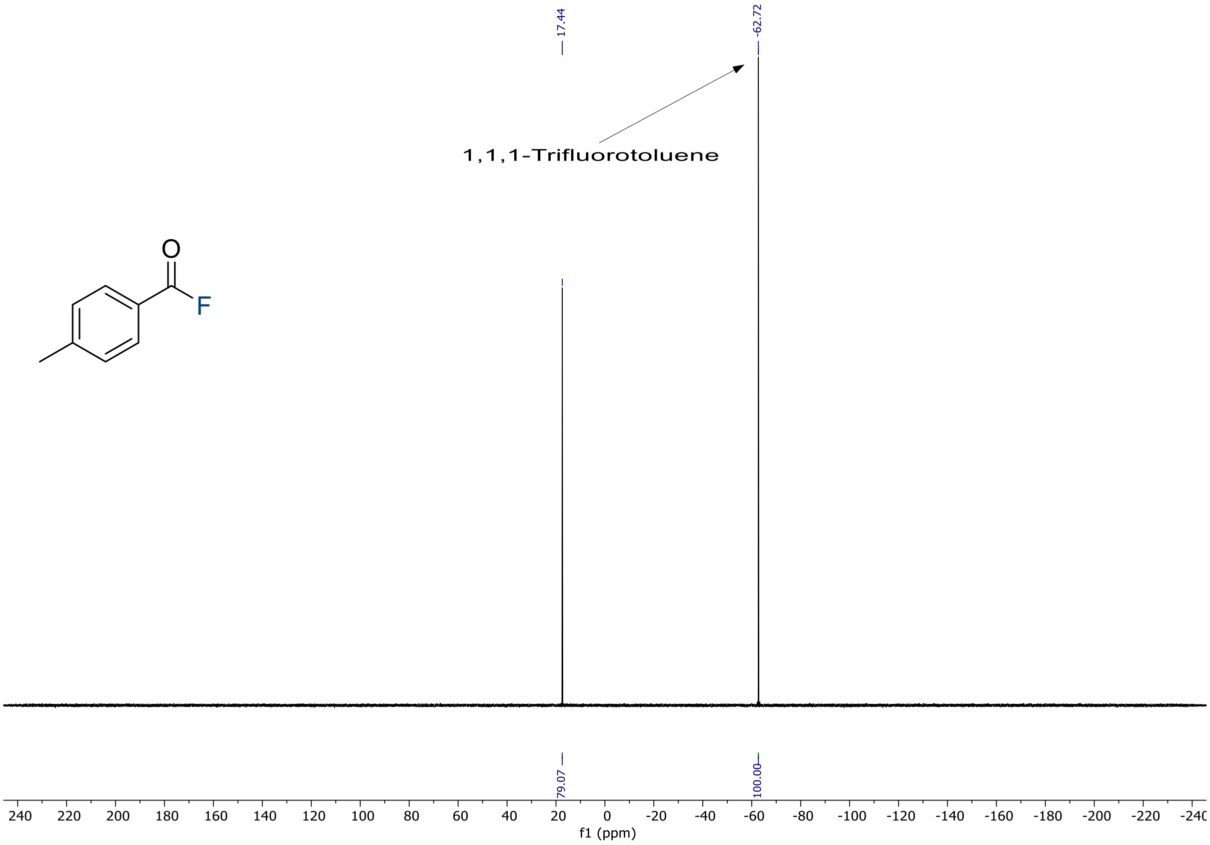

Crude, quantitative, ^{19}F NMR (282 MHz, $CDCl_3$, 298 K) spectrum of **1l** with 1,1,1-trifluorotoluene (0.33 eq.) added as internal standard.

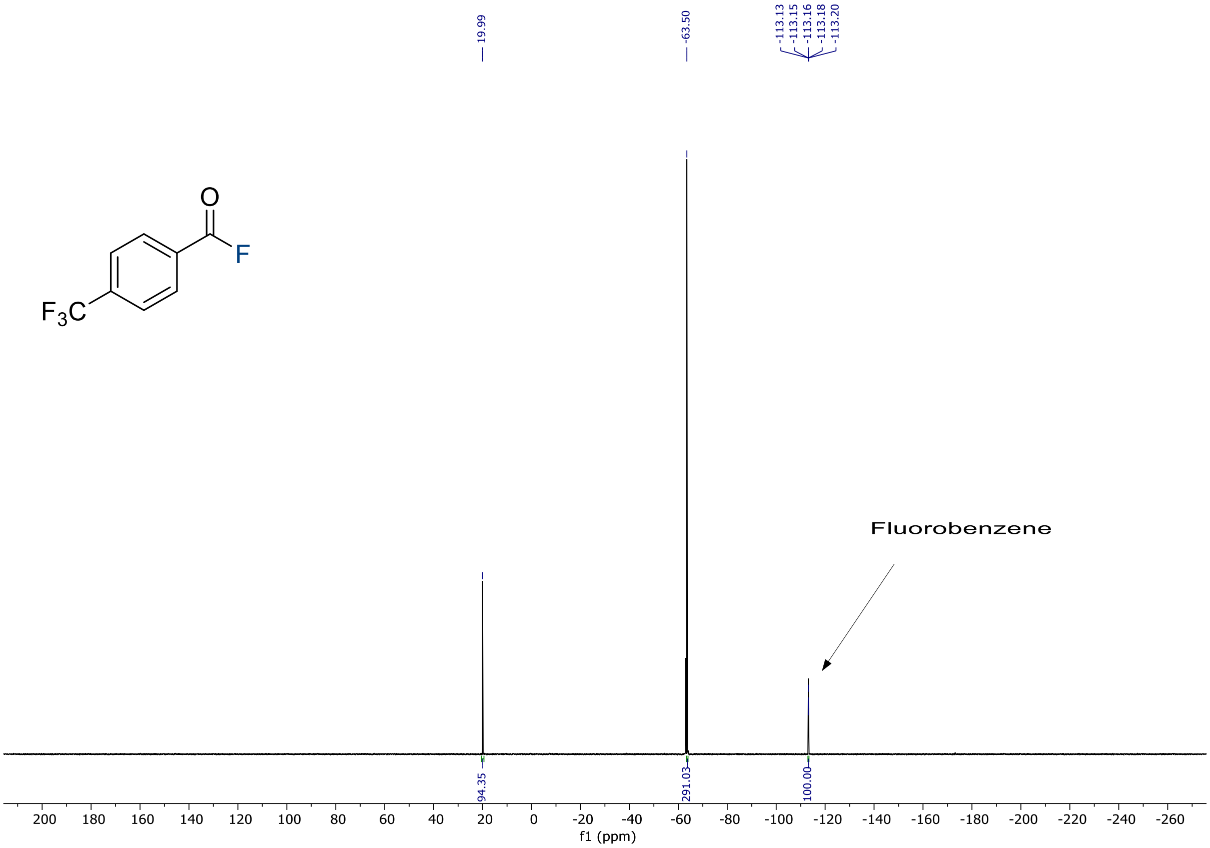

Crude, quantitative, ^{19}F NMR (282 MHz, $CDCl_3$, 298 K) spectrum of **1m** with fluorobenzene (1.00 eq.) added as internal standard.

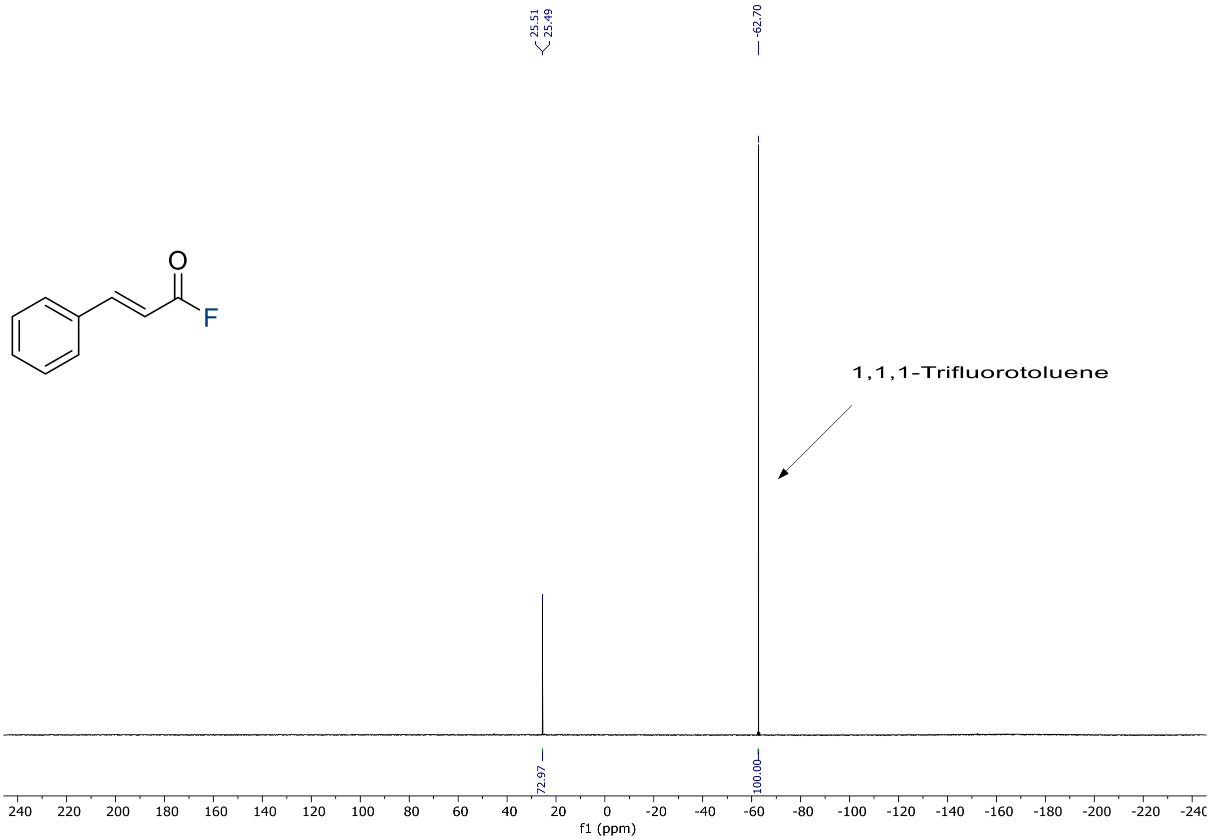

Crude, quantitative, ^{19}F NMR (282 MHz, $CDCl_3$, 298 K) spectrum of **1n** with 1,1,1-trifluorotoluene (0.33 eq.) added as internal standard.

5. References

(1) A. Sadoc, M. Body, C. Legein, M. Biswal, F. Fayon, X. Rocquefelte, F. Boucher, *Phys. Chem. Chem. Phys.* **2011**, *13*, 18539–18550.

(2) S. Hayashi, K. Hayamizu, *Bull. Chem. Soc. Jpn.* **1990**, *63*, 913–919.

(3) D. Louvel, A. Chelagha, J. Rouillon, P.-A. Payard, L. Khrouz, C. Monnereau, A. Tlili, *Chem. Eur. J.* **2021**, *27*, 8704–8708.

(4) Y. Liu, D. Yu, Y. Guo, J.-C. Xiao, Q.-Y. Chen, C. Liu, *Org. Lett.* **2020**, *22*, 2281–2286.

(5) X. Kong, Q. Liu, Y. Chen, W. Wang, H.-F. Chen, W. Wang, S. Zhang, X. Chen, Z.-Y. Cao, *Green Chem.* **2024**, 26, 3435–3440.

(6) C. Patel, E. André-Joyaux, J. A. Leitch, X. M. de Irujo-Labalde, F. Ibba, J. Struijs, M. A. Ellwanger, R. Paton, D. L. Browne, G. Pupo, S. Aldridge, M. A. Hayward, V. Gouverneur, *Science*, **2023**, *381*, 302–306.

(7) M. Pérez-Palau, J. Cornella, *Eur. J. Org. Chem.* **2020**, 2497–2500.

(8) X. Kong, Y. Chen, Q. Liu, W. Wang, S. Zhang, Q. Zhang, X. Chen, Y.-Q. Xu, Z.-Y. Cao, *Org. Lett.* **2023**, *25*, 581–586.

(9) S. Zhao, Y. Guo, Z. Su, C. Wu, W. Chen, Q.-Y. Chen, *Chin. J. Chem.* **2021**, *39*, 1225–1232.

(10) M. Arisawa, Y. Igarashi, H. Kobayashi, T. Yamada, K. Bando, T. Ichikawa, M. Yamaguchi, *Tetrahedron* **2011**, *67*, 7846–7859.

(11) W. D. G. Brittain, S. L. Cobb, *Org. Lett.* **2021**, *23*, 5793–5798.

(12) Y. Ogiwara, D. Sakino, Y. Sakurai, N. Sakai, *Eur. J. Org. Chem.* **2017**, 4324–4327.

(13) C. Bonnefoy, A. Gallego, C. Delobel, B. Raynal, M. Decourt, E. Chefdeville, G. Hanquet, A. Panossian, F. R. Leroux, F. Toulgoat, T. Billard, *Eur. J. Org. Chem.* **2024**, *27*, e202400142.
